# Design and Synthesis of a Novel PLK1 Inhibitor Scaffold Using a Hybridized 3D-QSAR Model

**DOI:** 10.3390/ijms22083865

**Published:** 2021-04-08

**Authors:** Youri Oh, Hoyong Jung, Hyejin Kim, Jihyun Baek, Joonhong Jun, Hyunwook Cho, Daseul Im, Jung-Mi Hah

**Affiliations:** College of Pharmacy and Institute of Pharmaceutical Science and Technology, Hanyang University, Ansan 426-791, Korea; apdlzld4477@hanyang.ac.kr (Y.O.); hyong@hanyang.ac.kr (H.J.); gpwls6121@hanyang.ac.kr (H.K.); jhb3534@hanyang.ac.kr (J.B.); jjh0328@hanyang.ac.kr (J.J.); lod0201@hanyang.ac.kr (H.C.); bestimda@hanyang.ac.kr (D.I.)

**Keywords:** polo-like kinase 1 (PLK1), pyrazole, quantitative structure-activity relationship, hybridization

## Abstract

Polo-like kinase 1 (PLK1) plays an important role in cell cycle progression and proliferation in cancer cells. PLK1 also contributes to anticancer drug resistance and is a valuable target in anticancer therapeutics. To identify additional effective PLK1 inhibitors, we performed QSAR studies of two series of known PLK1 inhibitors and proposed a new structure based on a hybridized 3D-QSAR model. Given the hybridized 3D-QSAR models, we designed and synthesized 4-benzyloxy-1-(2-arylaminopyridin-4-yl)-1*H*-pyrazole-3-carboxamides, and we inspected its inhibitory activities to identify novel PLK1 inhibitors with decent potency and selectivity.

## 1. Introduction

Polo-like kinases (PLKs) are a family of five serine/threonine kinases [1,2] that have been identified in various eukaryotic organisms and play important roles in cell proliferation, especially cell cycle regulation [3]. PLK1 is involved in the mitotic cell cycle, peaking in the S phase, and gradually decreasing in the G phase. Thus, PLK1 appears to induce cell proliferation, and the overexpression of PLK1 is common in malignant tumors. Among PLKs, PLK1 is overexpressed in a wide range of human cancers, including leukemia [4], and is considered an attractive anticancer drug target [5]. In contrast, since PLK2 (also known as SNK) [6] and PLK3 (also known as FNK or PRK) [7,8] are tumor suppressors, development of a PLK1 inhibitor with isoform selectivity is very important. In addition, PLK1 is involved in resistance [9] to several anticancer drugs such as doxorubicin, paclitaxel, and gemcitabine. This is presumed to be the mechanism by which PLK1 promotes the cell cycle and, directly or indirectly, inactivates p53, suggesting that the inhibition of PLK1 can be very useful in single and combination anticancer therapy.

**Figure 1:**
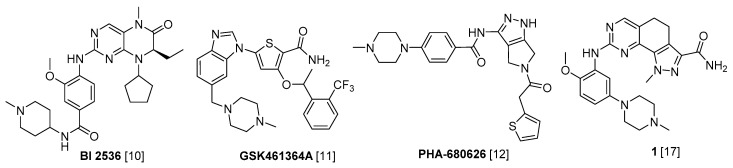
Representative PLK1 inhibitors.

These properties have led many groups to focus on the development of PLK1 inhibitors, and developed PLK1 inhibitors have been able to uncover new cellular functions of PLK1 in many laboratories. Among them, BI 2536, developed by Boehringer Ingelheim, is a potent ATP competitive PLK1 inhibitor with IC_50_ of 0.83 nM [10]. It also inhibits the activities of PLK2 (IC_50_ = 3.5 nM) and PLK3 (IC_50_ = 9.0 nM). BI 2536 showed moderate selectivity for inhibiting kinase activity of PLK1 among PLK members. The level of PLK1 is high in dividing cells, whereas PLK2 and PLK3 are not specifically expressed in proliferating cells. However, its weak selectivity to the isoforms of PLK1 was pointed out as a possible hurdle. Treatment of BI 2536 induced mitotic arrest in prometaphase, forming aberrant mitotic spindles and, consequently, apoptosis. When BI 2536 was used in human cancer cells, it inhibited cell proliferation in several human cancer cell lines, showing an effect on diverse organ derivatives such as breast, colon, lung, pancreas, and prostate cancer preclinical data showed that BI 2536 could be a possible anticancer drug candidate, leading to an investigation of the clinical effects of BI 2536. Monotherapy with BI 2536, the first human study of PLK1 inhibitors, has been terminated now, but its combinational study is still available in several solid tumors. In subsequent pharmaceutical efforts, new PLK1 inhibitors such as GSK461364A [11], and PHA-680626 [12] were all developed to inhibit PLK1 with low nanomolar IC_50_s and exert potent antiproliferative effects on various cancer cells. (Figure 1). Many of the PLK1 inhibitors discovered as a result of such studies have reached preclinical success and phase 1 clinical trials, but most have failed at this stage due to toxicity issues. Among them, PLK1 inhibitors such as BI 2536 [10] and GSK461364A [11] passed phase 1 clinical trials and progressed to phase 2 and phase 3 studies as treatment for specific malignancies. Nevertheless, the results of those studies were not as effective as expected when treatment was used as a second- or third-line therapy [13,14,15,16]. Only a small number of patients showed disease stabilization, indicating that further exploration of PLK1 inhibitors and related treatment plans is needed.

Medicinal chemists rely on understanding structure-activity relationships (SAR) for drug discovery and development. However, the SAR are mostly deduced from syntheses of numerous compounds and the establishment of bioactivity assay data. These conventional processes are essential but time-consuming and labor intensive. In contrast, in silico approaches deduce SAR by statistical analysis of known active compounds. Calculations convert SAR into 3D quantitative structure-activity relationships (3D-QSAR), which are more efficient than conventional SAR in terms of time and cost. The QSAR results indicate the properties of a ligand with increased activity, such as steric, electrostatic interactions; hydrogen bonding affinity; hydrophobicity. In that context, we performed QSAR studies of two known PLK1 inhibitor series and proposed a new structure based on hybridization of the two resulting QSAR models for discovery of a novel effective PLK1 inhibitor. Of known 3D-QSAR methods, Comparative Molecular Field Analysis (CoMFA) and Comparative Molecular Similarity Index Analysis (CoMSIA) are the most common, and we used them for model validation of a hybridized QSAR model from two series of PLK1 inhibitors in the literature. The hybrid 3D-QSAR model suggested a guide for new scaffolds, based on which we designed and synthesized 4-benzyloxy-1-(2-arylaminopyridin-4-yl)-1*H*-pyrazole-3-carboxamide. Using these approaches, we could identify a novel PLK1 inhibitor with decent potency and improved selectivity.

## 2. Results and Discussion

### Rational Design of a Hybridized 3D-QSAR Model

**Figure 2:**
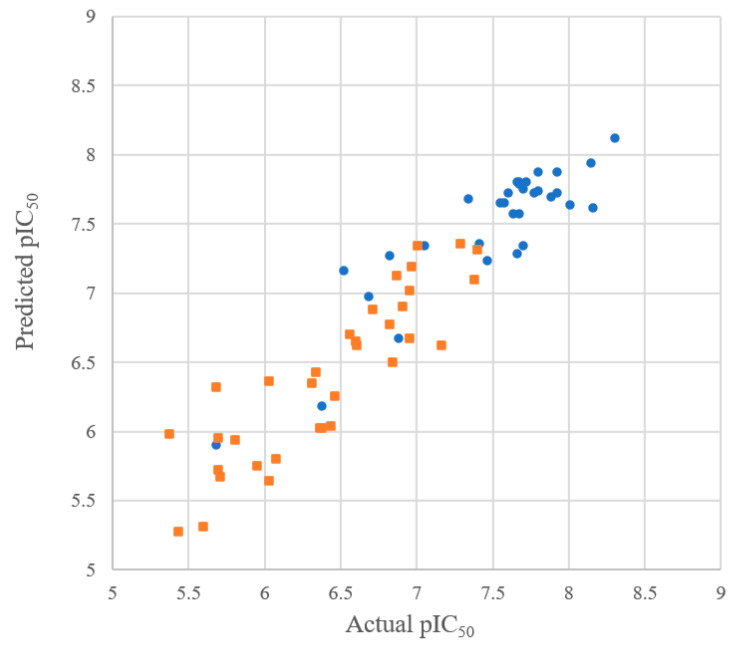
Observed vs. predicted values of the hybridized CoMFA model. 8-Amino-4,5-dihydro-1*H*-pyrazolo [4,3-*h*]quinazoline-3-carboxamides (series A; orange) and thiophene-2-carboxamide (series B; cyan).

**Figure 3:**
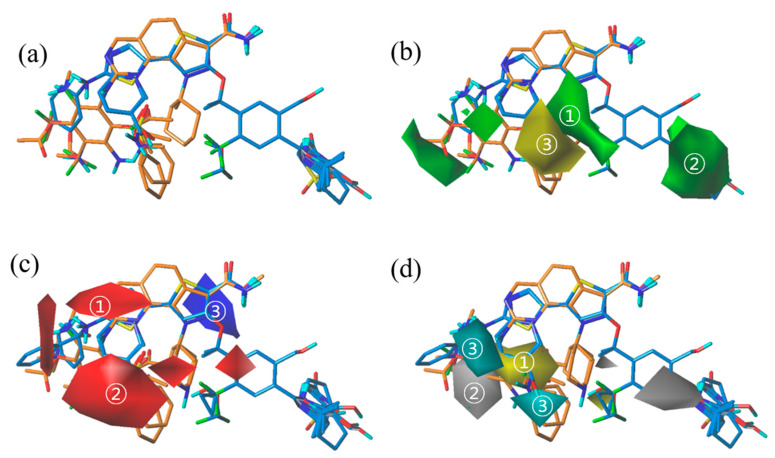
(**a**) Alignment of two series of compounds. Thiophene-2-carboxamide (cyan) and 8-amino-4, 5-dihydro-1*H*-pyrazolo[4,3-h]quinazoline-3-carbaldehyde derivatives (orange). (**b**) CoMFA steric contour map, (**c**) CoMFA electrostatic contour map, and (**d**) CoMSIA contour map.

First, we selected two series of PLK1 inhibitors, series A of 44 8-amino-4, 5-dihydro-1*H*-pyrazolo [4, 3-*h*] quinazoline-3-carboxamide derivatives [17] and series B of 36 thiophene-2-carboxamide derivatives [18]. Then, we sorted 66 of 80 compounds, excluding 10 with low potency (IC_50_ > 3 µM) and four racemates to establish the hybridized 3D-QSAR model. We used pIC_50_ (= −log IC_50_) value as the dependent variable in the QSAR model and divided the 66 compounds into six groups (Supplementary Section S1). As a test set in each section, two compounds per group were selected. The remaining 54 compounds, excluding the 12 of the test set, were used as the training set. Using these, we generated CoMFA models with Gasteiger–Hückel charged descriptor through SYBYL-X 2.1.1 automatic PLS (Figure 2). All the compounds were prepared in 3D conformation with SYBYL-X Ligand preparation based on ligands bound to PDB 3KB7 [13,14,15,16]. The two series of compounds overlapped two functional groups of each series, *thiophene-2-carboxamide* and *pyrazol-3-carboxamide* (Figure 3a). The CoMSIA model was generated through a similar process. We select steric, electrostatic, hydrophobic, hydrogen bond donor, and hydrogen bond acceptor indices as descriptors. The statistical parameters of the CoMFA and CoMSIA models are in Table 1.

We obtained steric and electrostatic contour maps from the CoMFA model. In Figure 3b, the green field is the bulkiness-favored zone, and yellow is the bulkiness-disfavored zone. The cyclohexyl substituent in pyrazole (① series A; orange) and the substituents at the 4 position of the benzyloxy thiophene ring (② series B; cyan) were preferred in the green zone. The substituents on the imidazo[1,2-a]pyridine ring (the series B; cyan) and the amino pyrimidine ring (the series A; orange) were scattered in the mixed green and yellow zone (③). To better construct the models, we employed suitable rings with various substituents at this position. On electrostatic contour maps (c), the electrostatic field was visualized using blue for positive charge-favored zones and red for negative charge-favored zones. The aligned thiophene-2-carboxamides (the series B; cyan) and pyrazole-3-carboxamides (series A; orange) required positive charges at the aminopyrimidine (①), piperazine substitution (②) at the aniline in pyrimidine, and negative charges at the bottom of the thiophene and pyrazole rings (③). Additionally, the CoMSIA contour map containing hydrophobic, hydrogen bond donor fields were generated (d). The two major hydrophobic spots (yellow) on the substituents of the imidazo[1,2-a]pyridine ring (①) were found, which were near the hydrophilic gray field, aminopyrimidine (②). The two hydrogen bond donor fields (cyan) are shown on the map near the imidazo[1,2-a]pyridine ring and piperazine substitution (③). No hydrogen bond disfavored-field was observed.

**Figure 4:**
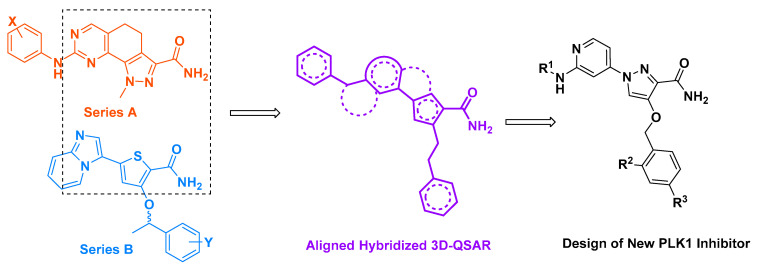
Design of a novel PLK1 inhibitor by the hybridization of two QSAR models.

From the hybridized CoMFA and CoMSIA models of the two series, several new chemical scaffolds were suggested by fusing unified alignments. First, we deduced a scaffold with a core, 1*H*-pyrrolo[2,3-c]pyridine ring with proper substituents satisfying the contour maps (Figure 3). Later, we modified the core, 1*H*-pyrrolo[2,3-c]pyridine ring into aminopyridine for flexibility, since some substituents were not properly positioned in hybridized CoMFA and CoMSIA models. The carboxamide group was unchanged, and the red fields in the electrostatic contour map (Figure 3c, ②) contributed to introducing aniline with electronegative substitutions to the pyridine ring and the 2-fluoro-4-trifluoromethyl benzene for the electronegative field. In the trimming step, we imposed bulky substituents (such as a dimethylamine) at perpendicular conformation of 2-carboxamide of the pyrazole, inducing the favored electrostatic field by rotation of the carboxamide group. Finally, we produced ***4-((2-R^2^-4-R^3^-benzyl)oxy)-1-(2-(2-R^1^-aminopyridin-4-yl)-1H-pyrazole-3-carboxamide*** as a novel PLK1 inhibitor scaffold (Figure 4).

**Scheme 1:**
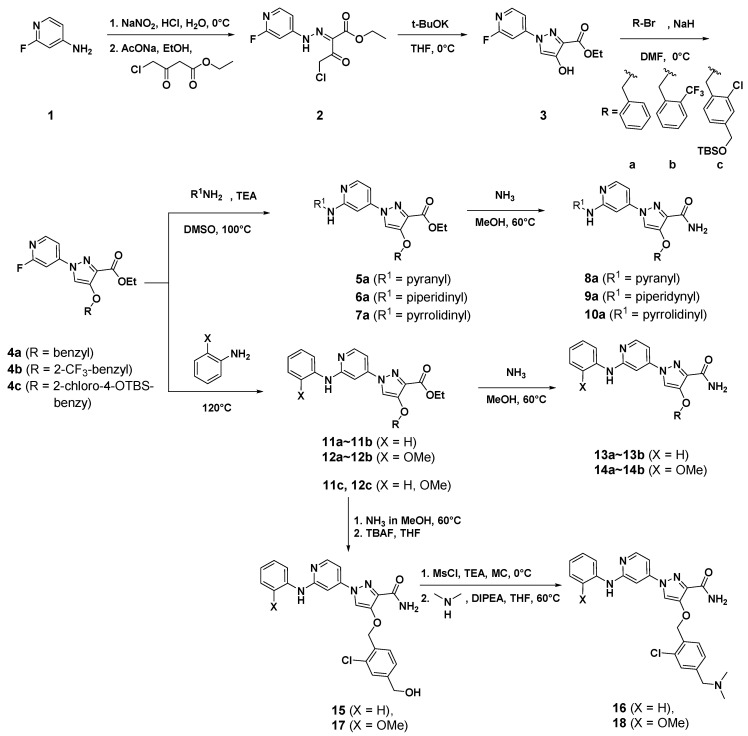
Synthesis of 4-((2-R^2^-4-R^3^-benzyloxy)-1-(2-(2-R^1^-amino)pyridin-4-yl)-1*H*-pyrazole-3-carboxamide.

The general synthesis of the designed 4-((2-R^2^-4-R^3^-benzyl)oxy)-1-(2-(2-R^1^-aminopyridin-4-yl)-1*H*-pyrazole-3-carboxamide (**8a**–**10a**, **13a**–**b**, **14a**–**b**, **15**–**18**) is shown in Scheme 1. For synthesis of the 1, 3, 4-substituted pyrazole core, we azo coupled diazotized 2-fluoropyridin-4-amine (**1**) and ethyl 4-chloro-3-oxobutanoate and obtained ethyl (E)-4-chloro-2-(2-(2-fluoropyridin-4-yl)hydrazinylidene)-3-oxobutanoate (**2**) as an intermediate. Subsequent cyclization using potassium *t*-butoxide resulted in a proper pyrazole core (**3**) [19], of which the hydroxyl group was alkylated with three types of benzyl bromide to yield **4a**–**4c**. Next, the fluorine of pyridine was substituted via S_N_Ar to give **5a**–**7a**, **11a**–**c**, and **12a**–**c** according to R^1^-NH_2_. Then, the 3-ethyl ester group was converted to carboxamide using ammonia in methanol to give **8a**–**10a**, **13a**–**b**, and **14a**–**b**. When the 2-chloro-4-*tert*- butyldimethylsilyl oxybenzyloxy group (synthesized in two steps from methyl 3-chloro-4-methylbenzoate; Supplementary S2) was employed as R^2^, further derivatizations were performed after conversion of ethyl ester (**11a**–**c**, **12a**–**c**) to desired product with benzylalcohol (**15**, **17**) and dimethylamino groups (**16**, **18**) by sequential deprotection and substitution.

**Figure 5:**
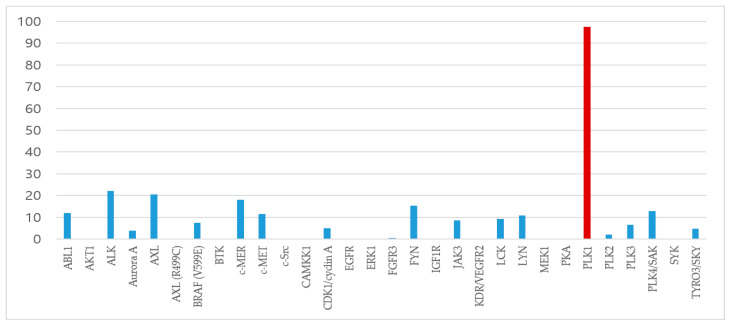
Percentages of enzymatic inhibition exerted by **15** (10 µM) on 30 selected protein kinases [21].

All the 1-pyridyl-4-substituted-pyrazole-3-carboxamide derivatives **8a**–**10a**, **13a**–**b**, **14a**–**b**, **and 15**–**18** were evaluated for inhibitory activity against PLK1 kinase, and the results are shown in Table 2. The synthesized compounds exhibited mild to potent inhibitory activity against PLK1, especially compounds that incorporated 2, 4-substituted benzyloxy moieties at the 4-position in the pyrazole core. Among the compounds evaluated, **15** showed the most potent activity against PLK1, with an IC_50_ value of 219 nM (Supplementary Section S3). We first noted that *ortho*-substituted phenyl is preferred as the R^1^ group on the pyridine ring to the cycloalkylamino group (**8a**, **9a**, **10a**). We suspect the aminopyridine group acts as a hydrogen bond-hinge binder, and the X substituent impacts the angles of the two hydrogen bonds. However, it is not clear whether 2-methoxy aniline or simple aniline is preferred from the value of IC_50_. Rather, the position of the bulky substituent on the pyrazole ring was found to play an important role in inhibitory activity. Specifically, pyrazole compounds incorporating a 2, 4-disubstituted benzyloxy group were 6–8 times more potent than compounds without substitution at the 2-position (**13a** vs. **13b**, **15**, **17**). Moreover, it seems that the hydroxyl substituent, rather than the dimethyl amino group, is preferred as R^3^ at the 4-position of the benzyloxy group, and the hydrogen bond, rather than the electrostatic interaction, could serve as an important interaction. For R^2^, a large substituent is clearly preferred to hydrogen, but there was not much difference between -CF_3_ and -Cl. We next performed a kinase panel screen for compound **15** against 30 protein kinases at 10 µM (Figure 5). Compound **15** achieved an excellent selectivity profile with an acquired inhibitory activity of 97% against PLK1 but with no significant activity against other protein kinases, especially PLK2 and PLK3. Additionally, compound **15** was not detected as PAINS [20]. Overall, this result indicates that the kinase activity profile and selectivity of the hybrid QSAR-driven inhibitor result in selective PLK1 inhibition, which could serve as a lead compound for the next step.

**Figure 6:**
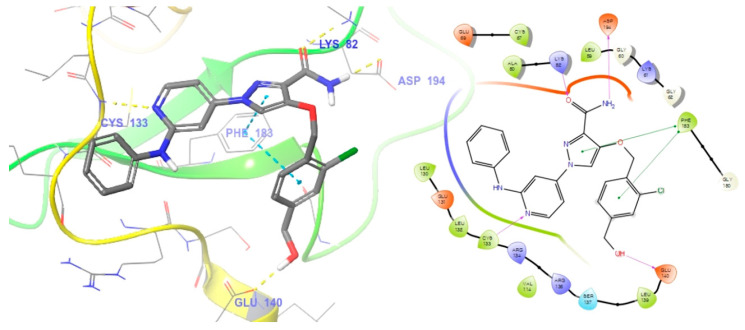
Docking structure of **15** at PLK1 (left, PDB: 3KB7) and the 2D-interaction map (right).

To better understand the interactions between the newly synthesized compounds and PLK1, we performed molecular docking studies of compound **15** in the ATP binding pocket of PLK1 (PDB: 3KB7) [13,14,15,16] using Glide (SCHRODINGER software package Version 14.2), and the results are shown in Figure 6. In binding mode, compound **15** was tightly bound to the ATP-binding site of PLK1 via several hydrogen bonds and π-π interactions. The most important interactions seemed to be a pair of hydrogen bonds between carboxamide and Lys82 or Asp194. Further, the nitrogen of pyridine, as a hinge binder, formed a hydrogen bond with the amino hydrogen of Cys 133. The pyrazole core and benzyl ring in the 3-benzyloxy group of **15** formed two π- π interactions with Phe184. In addition, the 4-methylenehydroxy group on the benzyloxy tail exhibited a hydrogen bond with Glu140, explaining the better potency compared to a simple benzyl group (vs. **13a**, **14a**).

## 3. Materials and Methods

### 3.1. QSAR Studies

From thiophene-2-carboxamide derivatives and 8-amino-4,5-dihydro-1*H*-pyrazolo[4,3-h]quinazoline derivatives, two representative compounds, **s18** and **s49** (Supplementary Materials), were selected as standards for the appropriate series. Then, **s18** and **s49** were matched by Fit Atom in SYBYL-X 2.1.1 to integrate the QSAR models. In this step, we used the conformations of **s18** and **s49** from a previous study [13,14,15,16]. The structures and activities toward PLK1 of 66 compounds are presented in the Supplementary Materials.

### 3.2. Chemistry

#### 3.2.1. General Chemical Methods

All chemicals were of reagent grade and were purchased from Aldrich (USA). Separation of the compounds by column chromatography was carried out with silica gel 60 (200–300 mesh ASTM, E. Merck, Germany). The quantity of silica gel used was 50–100 times the weight charged on the column. Thin layer chromatography (TLC) was run on silica gel-coated aluminum sheets (silica gel 60 GF254, E. Merck, Germany) and visualized under ultraviolet (UV) light (254 nm). Both ^1^H NMR and ^13^C NMR spectra were recorded on a Brucker model digital AVANCE III 400 MHz spectrometer at 25 °C using tetramethylsilane (TMS) as an internal standard. High-resolution MS (HR/MS) experiments were conducted with Q-TOF/Mass spectrometer 6530 (Agilent Technologies, Santa Clara, CA, USA) operated in positive-ion electrospray mode.

#### 3.2.2. Synthesis of Ethyl 1-(2-fluoropyridin-4-yl)-4-hydroxy-1*H*-pyrazole-3-carboxylate (**3**)

To a solution of 2-fluoropyridin-4-amine (0.776 mmol) in 1.35 mL of H_2_O and 0.206 mL (2.33 mmol) of 35% HCl at 0 °C, a solution of NaNO_2_ (0.776 mmol) and 0.2 mL of H_2_O was slowly added and reacted for 30 min. Then, a solution of ethyl 4-chloro-3-oxobutanoate (0.776 mmol) and 0.388 mL of EtOH was added to the reaction vessel, and NaOAc (4.66 mmol) was added, producing a solid. After stirring for 2 h, the organic layer was extracted with ethyl acetate and dried with anhydrous sodium sulphate (Na_2_SO_4_), and the solvent was evaporated to obtain compound **2**. Subsequently, compound **2** was dissolved in 0.776 mL of THF at 0 °C, and potassium t-butoxide (0.854 mmol) was added, followed by stirring for 1 h. After completion of the reaction, saturated NH_4_Cl solution was added, the organic layer was extracted with ethyl acetate and dried with anhydrous sodium sulphate (Na_2_SO_4_), and the solvent was evaporated, followed by column chromatography and purification under EA:Hex (1:3) conditions to obtain compound **3** (74%). **^1^H NMR** (400 MHz, DMSO-d_6_)) δ 9.58 (s, 1H), 8.32 (d, *J =* 5.7 Hz, 1H), 8.28 (d, *J =* 1.0 Hz, 1H), 7.84 (dd, *J =* 5.7, 1.3 Hz, 1H), 7.64 (d, *J =* 1.6 Hz, 1H), 4.32 (q, *J =* 7.1 Hz, 2H), 1.31 (t, *J =* 7.1 Hz, 3H).); **HRMS (ESI^+^)** m/z calculated for C_11_H_11_FN_3_O_3_ [M+H]+: 252.0779, found 252.0778.

#### 3.2.3. General Procedure A (**4a**–**4c**)

Compound **3** (0.0199 mmol) was dissolved in 0.995 mL of DMF at 0 °C, and NaH (0.0239 mmol) and the appropriate benzyl bromide (0.0199 mmol) were added, followed by stirring for 1 h. After completion of the reaction, the reaction mixture was worked up 6 times with ethyl acetate and brine. The organic layer was dried with anhydrous sodium sulphate (Na_2_SO_4_), and the solvent was evaporated to give compound **4.**

**4a** as yellow solid (98%): General procedure A was followed, using benzyl bromide and **3**^1^H NMR (400 MHz, DMSO-d_6_): **^1^H NMR** (400 MHz, DMSO) δ 8.81 (s, 1H), 8.37 (d, *J =* 5.7 Hz, 1H), 7.86 (d, *J =* 5.7 Hz, 1H), 7.65 (d, *J =* 1.7 Hz, 1H), 7.52–7.46 (m, 2H), 7.46–7.40 (m, 2H), 7.38 (dd, *J =* 5.0, 3.6 Hz, 1H), 5.12 (s, 2H), 4.32 (d, *J =* 7.1 Hz, 2H), 1.31 (t, *J =* 7.1 Hz, 3H).; **HRMS (ESI^+^)** m/z calculated for C_18_H_17_FN_3_O_3_ [M+H]+: 342.1248, found 342.1262.

**4b** (yellow solid, 92%): General procedure A was followed, using 1-(bromomethyl)-2-(trifluoromethyl)benzene and **3**.**^1^H NMR** (400 MHz, DMSO-d_6_) δ 8.89 (s, 1H), 8.38 (d, *J =* 5.7 Hz, 1H), 7.91 (dd, *J =* 12.4, 6.8 Hz, 2H), 7.80 (dd, *J =* 17.3, 7.9 Hz, 2H), 7.70 (d, *J =* 1.7 Hz, 1H), 7.62 (t, *J =* 7.8 Hz, 1H), 5.26 (s, 2H), 4.33 (q, *J =* 7.1 Hz, 2H), 1.30 (t, *J =* 7.1 Hz, 3H). **HRMS (ESI^+^)** m/z calculated for C_19_H_16_F_4_N_3_O_3_ [M+H]^+^ 410.1122, found 410.1111.

**4c** (yellow solid, 56%): General procedure A was followed, using ((4-(bromomethyl)-3-chlorobenzyl)oxy)(tert-butyl)dimethylsilane and **3**. **^1^H NMR** (400 MHz, DMSO-d_6_) δ 8.88 (s, 1H), 8.37 (d, *J =* 5.7 Hz, 1H), 7.88 (d, *J =* 5.7 Hz, 1H), 7.67 (d, *J =* 8.1 Hz, 2H), 7.44 (s, 1H), 7.35 (d, *J =* 7.9 Hz, 1H), 5.16 (s, 2H), 4.75 (s, 2H), 4.32 (t, *J =* 7.1 Hz, 2H), 1.31 (t, *J =* 7.1 Hz, 3H), 0.91 (s, 9H), 0.09 (s, 6H). **HRMS (ESI^+^)** m/z calculated for C_25_H_32_ClFN_3_O_4_Si [M+H]^+^: 520.1829, found 520.1789.

#### 3.2.4. General Procedure B (**5a**–**7a**)

Compound **4** (0.0293 mmol) was dissolved in 0.293 mL of DMSO, and the appropriate amine (0.0586 mmol) and TEA (0.0586 mmol) were added, followed by stirring at 100 °C for 24 h. After completion of the reaction, the reaction mixture was cooled to room temperature, and work up was performed 6 times with ethyl acetate and washed with brine. The organic layer was dried with anhydrous sodium sulphate (Na_2_SO_4_), and the solvent was evaporated, followed by column chromatography and purification under EA:Hex (1:1) conditions to obtain a compound.

**5a** (38%): General procedure B was followed, using tetrahydro-2*H*-pyran-4-amine and **4. ^1^H NMR** (400 MHz, CDCl_3_) δ 8.01 (d, *J =* 6.0 Hz, 1H), 7.55 (s, 1H), 7.45 (dd, *J =* 7.8, 1.1 Hz, 2H), 7.42–7.34 (m, 3H), 6.88 (d, *J =* 1.7 Hz, 1H), 6.76 (dd, *J =* 6.0, 2.0 Hz, 1H), 5.94 (s, 1H), 5.12 (s, 2H), 4.46 (q, *J =* 7.1 Hz, 2H), 4.01 (dt, *J =* 12.2, 3.9 Hz, 2H), 3.92 (s, 1H), 3.57 (td, *J =* 11.8, 2.3 Hz, 2H), 2.02 (s, 2H), 1.65–1.56 (m, 2H), 1.43 (t, *J =* 7.1 Hz, 3H).; **HRMS (ESI^+^)** m/z calculated for C_23_H_27_N_4_O_4_ [M+H]+: 423.2027, found 423.2129.

**6a** (37%): General procedure B was followed, using piperidin-4-amine and **4. ^1^H NMR** (400 MHz, DMSO-d_6_) δ 8.60 (s, 1H), 8.10 (d, *J =* 6.1 Hz, 1H), 7.48 (d, *J =* 6.9 Hz, 2H), 7.44–7.34 (m, 3H), 7.11 (d, *J =* 30.0 Hz, 2H), 5.10 (d, *J =* 8.8 Hz, 2H), 4.31 (q, *J =* 7.1 Hz, 2H), 3.82 (s, 1H), 3.62 (s, 2H), 1.91 (s, 2H), 1.78 (d, *J =* 13.1 Hz, 2H), 1.65 (s, 2H), 1.38 (s, 9H), 1.29 (d, *J =* 5.3 Hz, 3H).; **HRMS (ESI^+^)** m/z calculated for C_28_H_36_N_5_O_5_ [M+H]^+^: 522.2711, found 522.2722.

**7a** (53%): General procedure B was followed, using pyrrolidin-3-amine and **4. ^1^H NMR** (400 MHz, CDCl_3_) δ 8.06 (s, 1H), 7.55 (s, 1H), 7.47-7.43 (m, 2H), 7.42−7.33 (m, 3H), 6.82 (d, *J =* 12.8 Hz, 2H), 5.11 (s, 2H), 4.46 (q, *J =* 6.8 Hz, 2H), 4.42-4.34 (m, 1H), 3.73 (dd, *J =* 11.2, 6.0 Hz, 1H), 3.31 (s, 1H), 3.22 (s, 1H), 2.28–2.19 (m, 1H), 1.89 (d, *J =* 9.7 Hz, 2H), 1.46 (s, 9H), 1.42 (d, *J =* 7.1 Hz, 3H), 1.25-1.25 (m, 1H).; **HRMS (ESI^+^)** m/z calculated for C_27_H_34_N_5_O_3_ [M+H]^+^: 508.2554, found 508.2535.

#### 3.2.5. General Procedure C (**8a**–**10a**, **13a**–**13b**, **14a**–**14b**)

After adding 0.47 mL of 7N NH_3_ in MeOH to the appropriate ethyl 4-1*H*-pyrazole-3-carboxylate (0.0111 mmol), the mixture was stirred at 60 °C for 16 h. After completion of the reaction, the reaction mixture was cooled to room temperature and concentrated in vacuo. Column chromatography was performed and purified under MC:MeOH (20:1) conditions to obtain corresponding 4-1H-pyrazole-3-carboxamide.

**8a** as white solid (53%): General procedure C was followed, using **5a. ^1^H NMR** (400 MHz, DMSO-d_6_) δ 8.44 (s, 1H), 8.04 (d, *J =* 5.7 Hz, 1H), 7.53–7.45 (m, 3H), 7.45–7.39 (m, 2H), 7.38–7.33 (m, 1H), 7.25 (s, 1H), 6.95 (dd, *J =* 5.7, 2.0 Hz, 1H), 6.90 (d, *J =* 1.7 Hz, 1H), 6.75 (d, *J =* 7.7 Hz, 1H), 5.11 (s, 2H), 3.95 (d, *J =* 7.8 Hz, 1H), 3.86 (dd, *J =* 8.0, 3.3 Hz, 2H), 3.41 (td, *J =* 11.4, 2.1 Hz, 2H), 1.87 (d, *J =* 10.3 Hz, 2H), 1.49–1.40 (m, 2H); **^13^C NMR** (101 MHz, DMSO-d_6_) δ 161.7, 159.2, 149.3, 146.2, 145.5, 136.2, 128.5, 128.2, 128.0, 114.3, 101.1, 95.3, 73.5, 66.0, 46.4, 32.7 (Supplementary S5); mp: 78.5–80.5 °C; **HRMS (ESI^+^)** m/z calculated for C_21_H_24_N_5_O_3_ [M+H]+: 394.1874, found 394.1853.

**9a** (white solid, 30%): General procedure C was followed, using **6a. ^1^H NMR** (400 MHz, DMSO-d_6_) δ 8.82 (s, 2H), 8.50 (s, 1H), 8.11 (d, *J =* 5.7 Hz, 1H), 7.52-7.48 (m, 2H), 7.44–7.35 (m, 3H), 7.08 (dd, *J =* 5.8, 2.0 Hz, 1H), 6.97 (d, *J =* 1.7 Hz, 1H), 5.12 (s, 2H), 4.49–4.41 (m, 1H), 3.51–3.42 (m, 1H), 3.28-3.24 (m, 1H), 3.09 (dt, *J =* 11.0, 4.3 Hz, 1H), 2.23 (dt, *J =* 20.9, 7.3 Hz, 2H), 1.93 (dd, *J =* 15.9, 9.5 Hz, 2H); **^13^C NMR** (101 MHz, DMSO-d_6_) δ 161.6, 159.1, 149.2, 141.1, 145.5, 137.3, 136.7, 133.5, 127.3, 124.4, 121.3, 99.3, 88.9, 70.5, 36.4, 33.6; **mp**: 58.5–60.5 °C; **HRMS (ESI^+^)** calculated for C_21_H_25_N_6_O_2_ [M+H]+: 393.2034, found 393.2005.

**10a** (yellow solid, 57%): General procedure C was followed, using **7a. ^1^H NMR** (400 MHz, DMSO-d_6_) δ 8.48−8.43 (m, 1H), 8.06 (dd, *J =* 5.5, 3.6 Hz, 1H), 7.53−7.48 (m, 2H), 7.43–7.33 (m, 3H), 6.97 (d, *J =* 12.6 Hz, 1H), 6.85 (s, 1H), 5.11 (d, *J =* 5.8 Hz, 2H), 4.81−4.73 (m, 1H), 4.04 (s, 1H), 3.09 (s, 1H), 2.78 (s, 1H), 1.95 (d, *J =* 13.7 Hz, 2H), 1.47 (s, 2H), 1.30−1.25 (m, 2H); **^13^C NMR** (101 MHz, DMSO-d_6_) δ 161.6, 159.1, 157.9, 149.4, 146.3, 145.5, 136.3; mp: 59.0–61.0 °C; **HRMS (ESI^+^)** calculated for C_20_H_23_N_6_O_2_ [M+H]+: 379.1877, found 379.1848.

**13a** (brown solid, 73%): General procedure C was followed, using **11a. ^1^H NMR** (400 MHz, DMSO-d_6_) δ 9.27 (s, 1H), 8.53 (s, 1H), 8.24 (d, *J =* 5.7 Hz, 1H), 7.71–7.65 (m, 2H), 7.52 (dd, *J =* 6.9, 5.3 Hz, 3H), 7.45−7.40 (m, 2H), 7.39−7.34 (m, 1H), 7.31 (d, *J =* 1.6 Hz, 1H), 7.28 (dd, *J =* 8.5, 7.5 Hz, 3H), 7.22 (dd, *J =* 5.7, 2.0 Hz, 1H), 6.92 (t, *J =* 7.3 Hz, 1H), 5.14 (s, 2H) (Supplementary S4); ^13^C NMR (101 MHz, DMSO-d_6_) δ 161.6, 157.1, 149.2, 146.3, 145.6, 141.4, 136.7, 136.2, 128.6, 128.2, 128.0, 120.8, 118.3, 114.3, 103.5, 97.8, 73.5 (Supplementary S5); **mp**: 191 °C–193 °C; **HRMS (ESI^+^)** m/z calculated for C_22_H_20_N_5_O_2_ [M+H]^+^: 386.1612, found 386.1450.

**13b** (45%): General procedure C was followed, using **11b. ^1^H NMR** (400 MHz, DMSO-d_6_) δ 9.28 (s, 1H), 8.60 (s, 1H), 8.25 (d, *J =* 5.7 Hz, 1H), 7.89 (d, *J =* 7.6 Hz, 1H), 7.82 (d, *J =* 7.8 Hz, 1H), 7.77 (t, *J =* 7.6 Hz, 1H), 7.71–7.66 (m, 2H), 7.62 (t, *J =* 7.6 Hz, 1H), 7.53 (s, 1H), 7.32 (d, *J =* 1.6 Hz, 1H), 7.31−7.24 (m, 4H), 6.92 (t, *J =* 7.3 Hz, 1H), 5.28 (s, 2H); **^13^C NMR** (101 MHz, DMSO-d_6_) δ 161.6 (s), 157.1 (d, *J =* 9.1 Hz), 149.1 (s), 146.3 (s), 145.4 (s), 141.4 (s), 136.7 (s), 134.2 (s), 132.9 (s), 130.5 (s), 129.0 (s), 128.6 (s), 126.9 (s), 126.1 (d, *J =* 5.5 Hz), 120.8 (s), 118.3 (d, *J =* 10.3 Hz), 114.7 (s), 103.6 (s), 97.9 (s), 70.2 (s); **mp**: 188.5–190.5 °C; **HRMS (ESI^+^)** m/z calculated for C_23_H_19_F_3_N_5_O_2_ [M+H]^+^: 454.1485, found 454.1450.

**14a** (yellow solid 73%): General procedure C was followed, using **12a.** ^1^H NMR (400 MHz, DMSO-d_6_) δ 8.47 (s, 1H), 8.26 (s, 1H), 8.23-8.19 (m, 2H), 7.55–7.46 (m, 4H), 7.45–7.39 (m, 2H), 7.38 (dd, *J =* 5.0, 3.6 Hz, 1H), 7.28 (s, 1H), 7.22 (dd, *J =* 5.7, 1.9 Hz, 1H), 7.03 (dd, *J =* 8.0, 1.6 Hz, 1H), 6.97 (td, *J =* 7.7, 1.8 Hz, 1H), 6.93 (dd, *J =* 7.7, 1.7 Hz, 1H), 5.13 (s, 2H), 3.85 (s, 3H) (Supplementary S4); **^13^C NMR** (101 MHz, DMSO-d_6_) δ 161.7, 157.3, 149.0, 146.3, 145.6, 136.3, 129.8, 128.5, 128.2, 128.0, 121.9, 120.4, 120.0, 114.2, 110.9, 103.5, 98.2, 73.5, 55.7 (Supplementary S5); **mp**: 197.0–199.5 °C; **HRMS (ESI^+^)** m/z calculated for C_23_H_22_N_5_O_3_ [M+H]^+^: 416.1717, found 416.1683.

**14b** (white solid 67%): General procedure C was followed, using **12b. ^1^H NMR** (400 MHz, DMSO-d_6_) δ 8.53 (s, 1H), 8.24 (dd, *J =* 8.6, 2.6 Hz, 2H), 8.21 (d, *J =* 5.7 Hz, 1H), 7.89 (d, *J =* 7.7 Hz, 1H), 7.82 (d, *J =* 7.8 Hz, 1H), 7.77 (t, *J =* 7.6 Hz, 1H), 7.62 (t, *J =* 7.7 Hz, 1H), 7.51 (d, *J =* 5.7 Hz, 1H), 7.49 (d, *J =* 1.8 Hz, 1H), 7.30 (s, 1H), 7.26 (dd, *J =* 5.7, 1.9 Hz, 1H), 7.03 (dd, *J =* 8.0, 1.6 Hz, 1H), 6.97 (td, *J =* 7.7, 1.8 Hz, 1H), 6.92 (td, *J =* 7.6, 1.7 Hz, 1H), 5.28 (s, 2H), 3.86 (s, 3H); **^13^C NMR** (101 MHz, DMSO-d_6_) δ 161.7 (s), 157.2 (d, *J =* 10.4 Hz), 148.9 (d, *J =* 12.6 Hz), 146.3 (s), 145.4 (s), 136.5 (s), 134.2 (s), 132.9 (s), 130.5 (s), 129.8 (d, *J =* 7.2 Hz), 128.9 (s), 121.9 (s), 120.4 (s), 119.9 (d, *J =* 15.6 Hz), 114.6 (s), 110.9 (s), 103.7 (s), 98.4 (s), 70.2 (s), 55.7 (s). **mp**: 178.5–180.5 °C; **HRMS (ESI^+^)** m/z calculated for C_24_H_21_F_3_N_5_O_3_ [M+H]^+^: 484.1591, found 484.1546.

#### 3.2.6. General Procedure D (**11a**–**11c**, **12a**–**12c**)

Compound **4** (0.0293 mmol) was dissolved in the appropriate aniline (0.134 mL, 50 eq) and stirred at 120° C for 18 h. After completion of the reaction, the reaction mixture was cooled to room temperature, and work up was performed with ethyl acetate and water. The organic layer was dried with anhydrous sodium sulphate (Na_2_SO_4_) and the solvent was evaporated, followed by column chromatography and purification under EA:Hex (1:3.5) conditions to obtain product.

**11a** (62%): General procedure D was followed, using aniline and **4a ^1^H NMR** (400 MHz, DMSO-d_6_) δ 9.36 (s, 1H), 8.62 (s, 1H), 8.26 (d, *J =* 5.7 Hz, 1H), 7.70 (dd, *J =* 8.6, 1.0 Hz, 2H), 7.52–7.47 (m, 2H), 7.45–7.40 (m, 2H), 7.39–7.34 (m, 2H), 7.30–7.25 (m, 2H), 7.22 (dd, *J =* 5.8, 2.0 Hz, 1H), 6.92 (t, *J =* 7.3 Hz, 1H), 5.13 (s, 2H), 4.32 (q, *J =* 7.1 Hz, 2H), 1.31 (t, *J =* 7.1 Hz, 3H). **HRMS (ESI^+^)** m/z calculated for C_24_H_23_N_4_O_3_ [M+H]^+^: 415.1765, found 415.1821.

**11b** (42%): General procedure D was followed, using aniline and **4b. ^1^H NMR** (400 MHz, DMSO-d_6_) δ 9.37 (s, 1H), 8.71 (s, 1H), 8.27 (d, *J =* 5.7 Hz, 1H), 7.93 (d, *J =* 7.6 Hz, 1H), 7.79 (dd, *J =* 17.5, 7.9 Hz, 2H), 7.71 (dd, *J =* 8.6, 1.0 Hz, 2H), 7.61 (t, *J =* 7.6 Hz, 1H), 7.39 (d, *J =* 1.6 Hz, 1H), 7.31-7.25 (m, 3H), 6.92 (t, *J =* 7.3 Hz, 1H), 5.27 (s, 2H), 4.32 (q, *J =* 7.1 Hz, 2H), 1.30 (t, *J =* 7.1 Hz, 3H). **HRMS (ESI^+^)** m/z calculated for C_25_H_22_F_3_N_4_O_3_ [M+H]^+^: 483.1639, found 483.1793

**11c** (63%): General procedure D was followed, using aniline and **4c. ^1^H NMR** (400 MHz, DMSO-d_6_) δ 9.36 (s, 1H), 8.71 (s, 1H), 8.27 (d, *J =* 5.7 Hz, 1H), 7.73−7.69 (m, 2H), 7.67 (d, *J =* 7.9 Hz, 1H), 7.44 (s, 1H), 7.39 (d, *J =* 1.7 Hz, 1H), 7.35 (d, *J =* 7.8 Hz, 1H), 7.31–7.24 (m, 3H), 6.92 (t, *J =* 7.3 Hz, 1H), 5.17 (s, 2H), 4.75 (s, 2H), 4.32 (q, *J =* 7.1 Hz, 2H), 1.31 (t, *J =* 7.1 Hz, 3H), 0.91 (s, 9H), 0.09 (s, 6H). **HRMS (ESI^+^)** m/z calculated for C_31_H_38_ClN_4_O_4_Si [M+H]^+^: 593.2345, found 593.2464.

**12a** (79%): General procedure D was followed, using 2-methoxyaniline and **4a. ^1^H NMR** (400 MHz, DMSO-d_6_) δ 8.56 (s, 1H), 8.45 (s, 1H), 8.21 (dd, *J =* 6.7, 5.2 Hz, 2H), 7.54–7.47 (m, 3H), 7.45–7.39 (m, 2H), 7.36 (dt, *J =* 9.6, 4.3 Hz, 1H), 7.19 (dd, *J =* 5.7, 2.0 Hz, 1H), 7.03 (dd, *J =* 8.0, 1.6 Hz, 1H), 6.97 (td, *J =* 7.7, 1.7 Hz, 1H), 6.91 (td, *J =* 7.6, 1.6 Hz, 1H), 5.12 (s, 2H), 4.32 (q, *J =* 7.1 Hz, 2H), 3.85 (s, 3H), 1.31 (t, *J =* 7.1 Hz, 3H). **HRMS (ESI^+^)** m/z calculated for C_25_H_25_N_4_O_4_ [M+H]^+^: 445.1870, found 445.1954.

**12b** (48%): General procedure D was followed, using 2-methoxyaniline and **4b. ^1^H NMR** (400 MHz, DMSO-d_6_) δ 8.64 (s, 1H), 8.46 (s, 1H), 8.22 (dd, *J =* 5.5, 3.9 Hz, 2H), 7.93 (d, *J =* 7.7 Hz, 1H), 7.79 (dd, *J =* 17.2, 8.0 Hz, 2H), 7.61 (t, *J =* 7.6 Hz, 1H), 7.55 (dd, *J =* 4.9, 1.7 Hz, 1H), 7.24 (dd, *J =* 5.7, 2.0 Hz, 1H), 7.04–7.01 (m, 1H), 6.97 (td, *J =* 7.7, 1.8 Hz, 1H), 6.92 (td, *J =* 7.6, 1.6 Hz, 1H), 5.27 (s, 2H), 4.32 (q, *J =* 7.1 Hz, 2H), 3.85 (s, 3H), 1.30 (t, *J =* 7.1 Hz, 3H). **HRMS (ESI^+^)** m/z calculated for C_26_H_24_F_3_N_4_O_4_ [M+H]^+^: 513.1744, found 513.1765.

**12c** (81%): General procedure D was followed, using 2-methoxyaniline and **4c. ^1^H NMR** (400 MHz, DMSO-d_6_) δ 8.64 (s, 1H), 8.45 (s, 1H), 8.24−8.19 (m, 2H), 7.67 (d, *J =* 7.9 Hz, 1H), 7.54 (d, *J =* 1.8 Hz, 1H), 7.44 (s, 1H), 7.35 (d, *J =* 7.9 Hz, 1H), 7.23 (dd, *J =* 5.7, 2.0 Hz, 1H), 7.03 (dd, *J =* 8.0, 1.6 Hz, 1H), 6.97 (td, *J =* 7.7, 1.8 Hz, 1H), 6.94−6.89 (m, 1H), 5.17 (s, 2H), 4.75 (s, 2H), 4.32 (q, *J =* 7.1 Hz, 2H), 3.85 (s, 3H), 1.31 (t, *J =* 7.1 Hz, 3H), 0.91 (s, 9H), 0.09 (s, 6H). **HRMS (ESI^+^)** m/z calculated for C_32_H_40_ClN_4_O_5_Si [M+H]^+^: 623.2451, found 623.2416.

#### 3.2.7. 4-((2-chloro-4-(hydroxymethyl)benzyl)oxy)-1-(2-(phenylamino)pyridin-4-yl)-1*H*-pyrazole-3-carboxamide (**15**)

After adding 1.45 mL of 7 N NH_3_ in MeOH to **11c** (0.0244 mmol), the mixture was stirred at 60 °C for 24 h. After confirmation of completion of the reaction, the reaction mixture was cooled to room temperature and concentrated in vacuo to obtain compound ethyl 4-((4-(((tert-butyldimethylsilyl)oxy)methyl)-2-chlorobenzyl)oxy)-1-(2-(phenylamino)pyridin-4-yl)-1H-pyrazole-3-carboxylate. Next, the product (0.0244 mmol) was dissolved in 0.244 mL of THF, and teterabutylammonium fluoride 1M in THF (0.0244 mmol) was slowly added. After the reaction, work up was performed with ethyl acetate and saturated NH_4_Cl solution. The organic layer was dried with anhydrous sodium sulphate (Na_2_SO_4_), and the solvent was evaporated, followed by column chromatography and purification under MC:MeOH (40:1) conditions to obtain compound **15** (60%): **^1^H NMR** (400 MHz, DMSO-d_6_) δ 9.27 (s, 1H), 8.61 (s, 1H), 8.24 (d, *J =* 5.7 Hz, 1H), 7.68 (dd, *J =* 8.6, 1.0 Hz, 2H), 7.63 (d, *J =* 7.9 Hz, 1H), 7.54 (s, 1H), 7.46 (s, 1H), 7.35−7.31 (m, 2H), 7.30−7.22 (m, 4H), 6.92 (t, *J =* 7.3 Hz, 1H), 5.35 (t, *J =* 5.8 Hz, 1H), 5.19 (s, 2H), 4.52 (d, *J =* 5.8 Hz, 2H) (Supplementary S4); **^13^C NMR** (101 MHz, DMSO-d_6_) δ 161.6, 157.2, 149.2, 146.3, 145.3, 141.4, 136.6, 132.5, 131.7, 130.2, 128.7, 127.0, 125.1, 124.2, 120.8, 118.3, 114.5, 103.6, 97.9, 70.9, 61.9 (Supplementary S5); **mp**: 179.5–180.5°C; **HRMS (ESI^+^)** m/z calculated for C_23_H_21_ClN_5_O_5_ [M+H]^+^: 450.1327, found 450.1294.

#### 3.2.8. 4-((2-chloro-4-(hydroxymethyl)benzyl)oxy)-1-(2-((2-methoxyphenyl)amino) pyridin -4-yl)-1*H*-pyrazole-3-carboxamide (**17**)

Compound **17** was prepared by an analogous procedure as described for the preparation of **15**. (67%): **^1^H NMR** (400 MHz, DMSO-d_6_) δ 8.54 (s, 1H), 8.27−8.19 (m, 3H), 7.63 (d, *J =* 7.9 Hz, 1H), 7.53 (s, 1H), 7.49 (d, *J =* 1.8 Hz, 1H), 7.46 (d, *J =* 1.3 Hz, 1H), 7.33 (d, *J =* 7.8 Hz, 1H), 7.30–7.23 (m, 2H), 7.03 (dd, *J =* 7.9, 1.6 Hz, 1H), 6.99-6.94 (m, 1H), 6.92 (dd, *J =* 7.5, 5.9 Hz, 1H), 5.35 (t, *J =* 5.8 Hz, 1H), 5.19 (s, 2H), 4.52 (d, *J =* 5.8 Hz, 2H), 3.86 (s, 3H) (Supplementary S4). **^13^C NMR** (101 MHz, DMSO-d_6_) δ 161.6, 157.2, 149.2, 146.3, 145.4, 141.4, 137.2, 136.6, 132.4, 130.1, 129.4, 128.6, 120.8, 118.3, 118.3, 114.6, 103.6, 97.9, 70.9, 44.8, 29.0. **mp**: 198.5-200.5 °C; **HRMS (ESI^+^)** m/z calculated for C_24_H_23_ClN_5_O_4_ [M+H]^+^: 480.1433, found 480.1406.

#### 3.2.9. General Procedure E (**16**, **18**)

After dissolving compound **15** (9.56 mmol) in 0.1 mL of MC, triethylamine (10.5 mmol) and methanesulfonyl chloride (14.3 mmol) were sequentially added dropwise at 0 °C and stirred for 3 h. After the reaction was complete, the organic layer was extracted using water and methylene chloride. The organic layer was dried with anhydrous sodium sulfate, the solvent was evaporated, and the next reaction proceeded. The obtained compound (0.00956 mmol) was dissolved in 0.1 mL of THF, and dimethylamine (19.1 mmol) and DIPEA (19.1 mmol) were added, followed by stirring at 60 °C for 18 h. After completion of the reaction, the mixture was cooled to room temperature, and work up was performed with ethyl acetate and water. The organic layer was dried with anhydrous sodium sulphate, and the solvent was evaporated, followed by column chromatography and purification under MC:MeOH (10:1) to obtain compound **16** as white solid (50%): **^1^H NMR** (400 MHz, DMSO-d_6_) δ 9.27 (s, 1H), 8.61 (s, 1H), 8.24 (d, *J =* 5.7 Hz, 1H), 7.71−7.66 (m, 2H), 7.63 (d, *J =* 7.8 Hz, 1H), 7.54 (s, 1H), 7.44 (s, 1H), 7.34-7.31 (m, 2H), 7.30–7.23 (m, 4H), 6.92 (t, *J =* 7.3 Hz, 1H), 5.19 (s, 2H), 3.42 (s, 2H), 2.16 (s, 6H) (Supplementary S4). **^13^C NMR** (101 MHz, DMSO-d_6_) δ 161.6, 157.2, 149.2, 146.3, 145.4, 141.4, 137.2, 136.6, 132.4, 130.1, 129.4, 128.6, 120.8, 118.3, 118.3, 114.6, 103.6, 97.9, 70.9, 44.8, 29.0; **mp**: 138.5–140.5 °C; **HRMS (ESI^+^)** m/z calculated for C_25_H_26_ClN_6_O_2_ [M+H]^+^: 477.1800, found 477.1767.

**18(.** white solid, 39%): Compound **18** was prepared by the same procedure as described for the preparation of **16** using **17. ^1^H NMR** (400 MHz, DMSO-d_6_) δ 8.54 (s, 1H), 8.27–8.19 (m, 3H), 7.63 (d, *J =* 7.8 Hz, 1H), 7.54–7.48 (m, 2H), 7.44 (d, *J =* 1.3 Hz, 1H), 7.32 (d, *J =* 7.8 Hz, 1H), 7.29 (s, 1H), 7.25 (dd, *J =* 5.7, 1.9 Hz, 1H), 7.03 (dd, *J =* 7.9, 1.6 Hz, 1H), 6.97 (td, *J =* 7.7, 1.8 Hz, 1H), 6.92 (td, *J =* 7.6, 1.7 Hz, 1H), 5.18 (s, 2H), 3.86 (s, 3H), 3.41 (s, 2H), 2.15 (s, 6H) (Supplementary S4); **^13^C NMR** (101 MHz, DMSO-d_6_) δ 161.7, 157.2, 148.9, 145.9, 136.4, 132.4, 132.1, 130.1, 129.8, 129.3, 127.6, 121.9, 120.4, 119.9, 114.5, 110.9, 103.6, 98.3, 70.9, 62.2, 55.7, 44.9; **mp**: 160–162 °C; **HRMS (ESI^+^)** m/z calculated for C_26_H_28_ClN_6_O_3_ [M+H]^+^: 507.1906, found 507.1881.

### 3.3. Molecular Modelling

Compounds were docked into the PLK1 structure (PDB: 3KB7) [13,14,15,16]. Protein and ligand preparations were performed with Schrödinger’s tools at standard settings, and Glide was used for docking and scoring. The 3D X-ray protein structures of PLK1 wildtype as a complex with a ligand were obtained from the PDB (code: 3KB7) and prepared using the Protein Preparation Wizard of the Schrödinger Maestro program. All water molecules were removed from the structure, and it was selected as a template. The structures of inhibitors were drawn using Chemdraw, and their 3D conformation was generated using the Schrödinger LigPrep program with the OPLS 2005 force field. Molecular docking of compound into the structure of PLK1 wildtype (PDB code: 3KB7) was carried out using Schrodinger Glide Version 12.2 (Schrödinger, LLC, New York, NY, USA).

### 3.4. Evaluation of IC_50_ Values and Selected Kinase Profiling

We used Reaction Biology Corp. Kinase Hot Spot^SM^ service (www.reactionbiology.com, (accessed on 15 December 2020)) for screening of **15** (10 µM) and IC_50_ Profiler Express for IC_50_ measurement. Assay protocol: In a final reaction volume of 25 μL, substrate[Casein], 1 μM, ATP 10 μM, PLK1(h) (5–10 mU) is incubated with 25 mM Tris pH 7.5, 0.02 mM EGTA, 0.66 mg/mL myelin basic protein, 10 mM Mg acetate, and [^33^P-ATP] (specific activity approx. 500 cpm/pmol, concentration as required). The reaction is initiated by addition of the Mg-ATP mix. After incubation for 40 min at room temperature, the reaction is stopped by addition of 5 μL of a 3% phosphoric acid solution. Next, 10 μL of the reaction is spotted onto a P30 filtremat and washed three times for 5 min in 75 mM phosphoric acid and once in methanol prior to drying and scintillation counting. The 30 selected protein kinases were ABL1, AKT1, ALK, Aurora A, AXL, AXL (R499C), BRAF (V599E), BTK, c-MER, c-MET, c-Src, CAMKK1, CDK1/cyclin A, EGFR, ERK1, FGFR3, FYN, IGF1R, JAK3, KDR/VEGFR2, LCK, LYN, MEK1, PKA, PLK1, PLK2, PLK3, PLK4/SAK, SYK, and TYRO3/SKY**4.**

## 4. Conclusions

In conclusion, two series of PLK1 inhibitors were aligned and combined using the CoMFA and CoMSIA 3D QSAR models, and several novel chemical scaffolds were suggested. We designed and synthesized a series of 4-benzyloxy-1-(2-(aryl/alkylamino)pyridin-4-yl)-1*H*-pyrazole-3-carboxamide derivatives based on the hybridized QSAR models. Of the suggested analogues, we synthesized 11 compounds and tested for inhibitory activity against PLK1. Compound **15**, i.e., 4-((2-chloro-4-(hydroxymethyl)benzyloxy)-1-(2-(phenylamino)pyridin-4-yl)-1*H*-pyrazole-3-carboxamide, displayed the most potent inhibitory activity against PLK1, with an IC_50_ of 219 nM.

We successfully discovered a novel scaffold of PLK1 inhibitors by hybridizing two chemical series into a QSAR model. In addition, we performed a kinase panel screen using compound **15** for 30 kinases at a single dose of 10 µM in duplicate (Figure 5). The results of the screen showed that the newly synthesized PLK1 inhibitor had excellent selectivity profiles toward PLK1. Considering that PLK1 is significantly associated with various cancers, the unique chemical scaffold described in this study will be valuable for developing new molecules as potential therapeutic agents for this disease. Indeed, the above findings provide a theoretical basis for further structural modification of 4-benzyloxy-1-(2-(aryl/alkylamino)pyridin-4-yl)-1*H*-pyrazole-3-carboxamide derivatives as PLK1 inhibitors, and compound **15** is a promising lead for new therapeutics targeting cancer due to its strong kinase selectivity profile.

## Figures and Tables

**Table 1:** PLS results of final CoMFA and CoMSIA models.

Components		Receptor-Guided Aligned Model with MC Searching	
		CoMFA	CoMSIA
PLS statistics	q^2^	0.657	0.641
	r^2^	0.899	0.838
	N	3	3
	SEE	0.348	0.335
	*F*-value	125.847	125.847
	Predictive r^2^	0.899	0.838
Field contribution	Steric	0.396	0.044
	Electrostatic	0.604	0.266
	Hydrophobic	-	0.262
	Donor	-	0.158
	Acceptor	-	0.270

q^2^, LOO cross-validated correlation coefficient; r^2^, non-cross-validated correlation coefficient; n, number of components used in the PLS analysis; SEE, standard error estimate; *F* value, *F*-statistic for the analysis (Supplementary Tables S1 and S2)

**Table 2:**
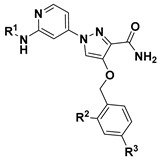
Enzymatic activities of 4-benzyloxy-1-(2-(aryl/alkylamino)pyridin-4-yl)-1*H*-pyrazole-3-carboxamide.

	R^1^	R^2^	R^3^	PLK1 IC_50_ (μM)
**8a**	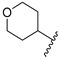	H	H	>10
**9a**	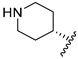			>10
**10a**	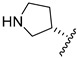			>10
**13a**	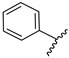	H	H	9.89
**13b**		CF_3_	H	0.847
**14a**	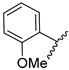	H	H	1.02
**14b**		CF_3_	H	1.31
**15**	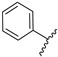	Cl	OH	0.219
**16**		Cl	N(Me)_2_	1.01
**17**	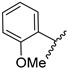	Cl	OH	0.312
**18**		Cl	N(Me)_2_	0.998
Control	Staurosporine			0.143

## Data Availability

Data is contained within the article and Supplementary Materials.

## Supplementary Materials

The following are available online at https://www.mdpi.com/article/10.3390/ijms22083865/s1.
