# Supplementary material for: Design and Synthesis of a Novel PLK1 Inhibitor Scaffold Using a Hybridized 3D-QSAR Model
Source: Int J Mol Sci. 2021 Apr 8;22(8):3865. doi: 10.3390/ijms22083865 (PMC8068361; doi:10.3390/ijms22083865)

Supplementary Materials

Design and synthesis of a novel PLK1 inhibitor scaffold *via* hybridized 3D-QSAR model

Youri Oh†, Hoyong Jung†, Hyejin Kim, Jihyun Baek, Joonhong Jun, Hyunwook Cho, Daseul Im and Jung-Mi Hah*

Department of Pharmacy, College of Pharmacy, Hanyang University 55 Hanyangdaehak-ro, Sangnok-gu, Ansan Kyeonggi-do, 426-791, Republic of Korea

S1. The structures of the chemically named compounds in QSAR studies

1) Thiophene-2-carboxamide derivatives

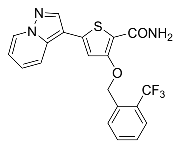

1

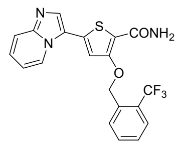

2

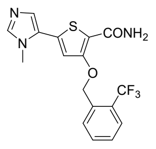

3

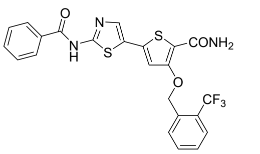

4

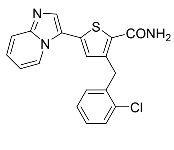

5

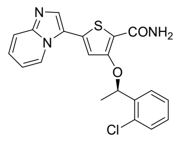

6

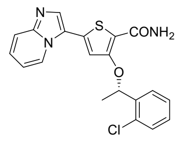

7

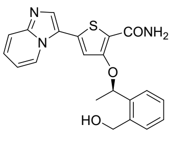

8

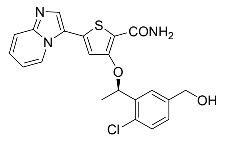

9

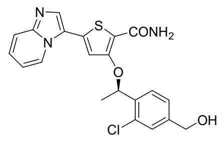

10

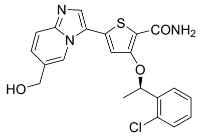

11

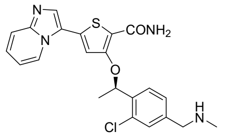

12

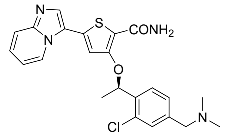

13

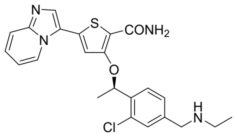

14

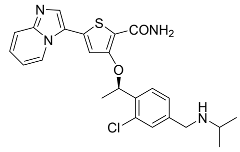

15

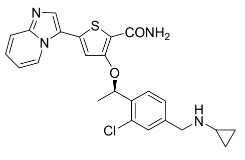

16

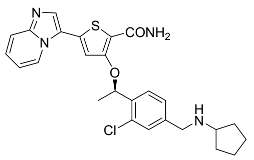

17

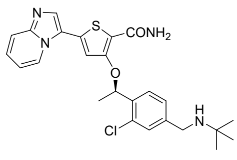

18

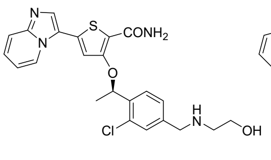

19

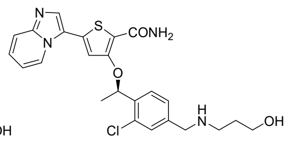

20

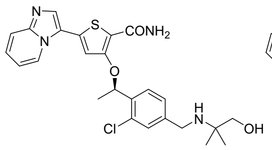

21

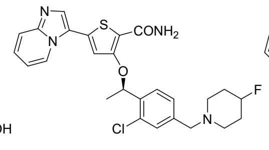

22

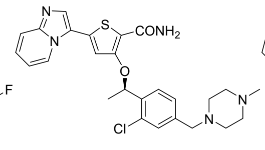

23

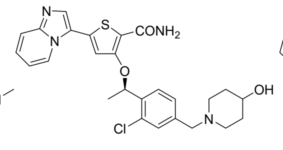

24

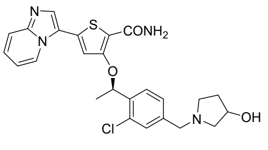

25

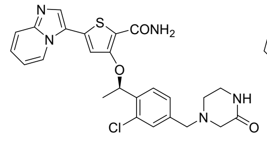

26

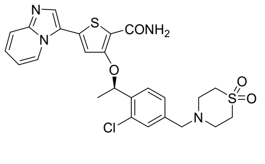

27

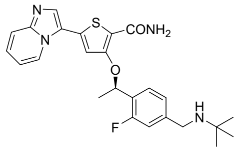

28

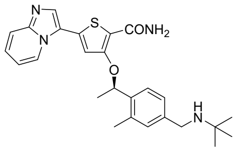

29

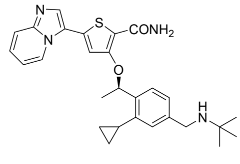

30

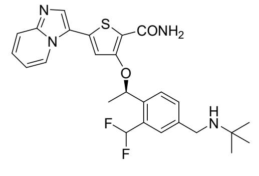

31

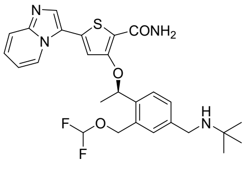

32

2) 8-Amino-4,5-dihydro-1*H*-pyrazolo[4,3-h]quinazoline-3-carboxamide derivatives

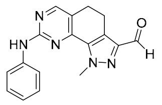

33

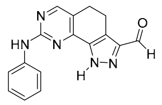

34

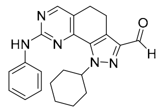

35

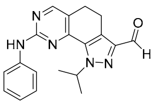

36

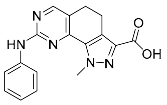

37

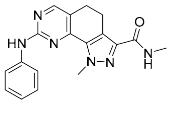

38

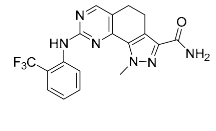

39

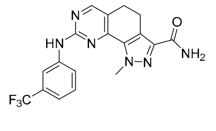

40

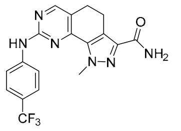

41

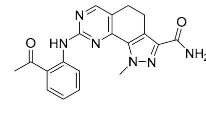

42

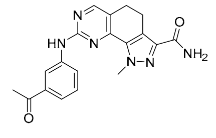

43

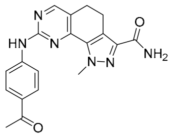

44

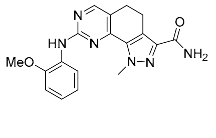

45

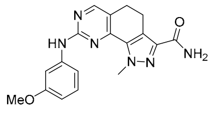

46

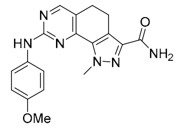

47

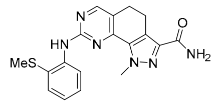

48

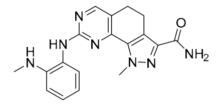

49

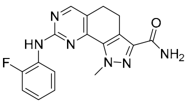

50

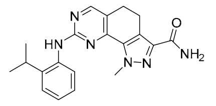

51

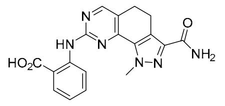

52

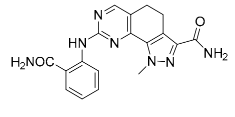

53

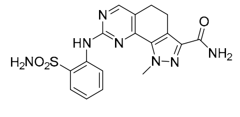

54

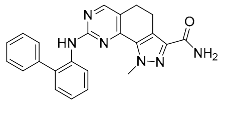

55

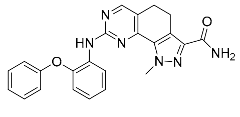

56

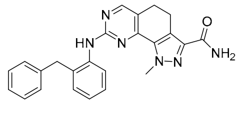

57

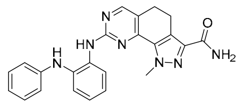

58

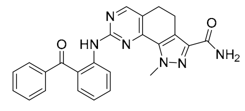

59

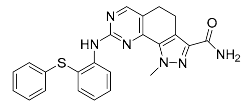

60

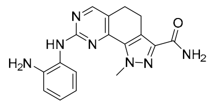

61

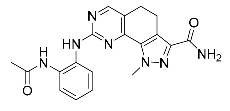

62

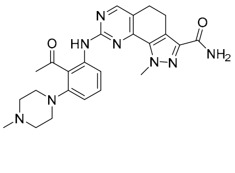

63

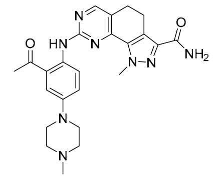

64

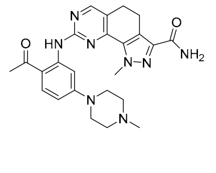

65

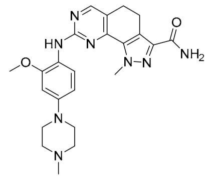

66

Alignments for CoMFA and CoMSIA

We obtained 36 thiophene-2-carboxamide derivatives and 44 8-amino-4,5-dihydro-*1H*-pyrazolo[4,3-h]quinazoline-3-carboxamide derivatives from the literature, and two representative compounds **18** and **49** were selected for standard compounds in each series. We excluded 12 compounds due to low activity (IC_{50} > 3 μM) and 5 that were racemates and outliers of the QSAR model. Finally, we sorted 66 compounds for the QSAR model. We used pIC_{50} values as the dependent variable in the QSAR model. The 66 compounds were split into a training set of 54 compounds to create a QSAR model and a test set of 12 compounds to validate the model. We used 1:6 ratio to divide the dataset compounds and also mention number of compounds selected in the test set based on the structure and activity (pIC50). This is also supported by saying that the test set compounds are selected in a way that they comprise compounds having high, moderate and low activity values. We used one of the algorithms given in the article to divide the dataset compounds into training and test sets, using Algorithm 4 (activity ranking).(*Journal of Computer-Aided Molecular Design*, 16: 357–369, 2002)

Table S1. The structures of thiophene-2-carboxamide derivatives and their activities on Plk1.

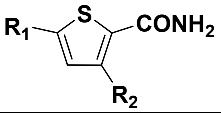

No.	Substiteuents		Activity (nM)	
	R1	R2	IC_{50}	pIC_{50}
1	pyrazolo[1,5-*a*]pyridin-3-yl	(2-(trifluoromethyl)benzyl)oxy	130	6.8861
2	imidazo[1,2-*a*]pyridin-3-yl	(2-(trifluoromethyl)benzyl)oxy	22	7.6576
3	1-methyl-1*H*-imidazol-5-yl	(2-(trifluoromethyl)benzyl)oxy	430	6.3665
4	2-benzamidothiazol-5-yl	(2-(trifluoromethyl)benzyl)oxy	2100	5.6778
5	imidazo[1,2-*a*]pyridin-3-yl	2-chlorobenzyl	35	7.4559
6	imidazo[1,2-*a*]pyridin-3-yl	(*R*)-1-(2-chlorophenyl)ethoxy	7	8.1549
7	imidazo[1,2-*a*]pyridin-3-yl	(*S*)-1-(2-chlorophenyl)ethoxy	300	6.5229
8	imidazo[1,2-*a*]pyridin-3-yl	(*R*)-1-(2-(hydroxymethyl)phenyl)ethoxy	88	7.0555
9	imidazo[1,2-*a*]pyridin-3-yl	(*R*)-1-(2-chloro-5-(hydroxymethyl)phenyl)ethoxy	39	7.4089
10	imidazo[1,2-*a*]pyridin-3-yl	(*R*)-1-(2-chloro-4-(hydroxymethyl)phenyl)ethoxy	4.9	8.3098
11	6-(hydroxymethyl)imidazo[1,2-*a*]pyridin-3-yl	(*R*)-1-(2-chlorophenyl)ethoxy	7.3	8.1367
12	imidazo[1,2-*a*]pyridin-3-yl	(*R*)-1-(2-chloro-4-((methylamino)methyl)phenyl)ethoxy	16	7.7959
13	imidazo[1,2-*a*]pyridin-3-yl	(*R*)-1-(2-chloro-4-((dimethylamino)methyl)phenyl)ethoxy	22	7.6576
14	imidazo[1,2-*a*]pyridin-3-yl	(*R*)-1-(2-chloro-4-((ethylamino)methyl)phenyl)ethoxy	21	7.6778
15	imidazo[1,2-*a*]pyridin-3-yl	(*R*)-2-chloro-4-((isopropylamino)methyl)phenyl)ethoxy	28	7.5528
16	imidazo[1,2-*a*]pyridin-3-yl	(*R*)-2-chloro-4-((cyclopropylamino)methyl)phenyl)ethoxy	12	7.9208
17	imidazo[1,2-*a*]pyridin-3-yl	(*R*)-2-chloro-4-((cyclopentylamino)methyl)phenyl)ethoxy	25	7.6021
18	imidazo[1,2-*a*]pyridin-3-yl	(*R*)-1-(4-((tert-butylamino)methyl)-2-chloropheny)ethoxy	21	7.6778
19	imidazo[1,2-*a*]pyridin-3-yl	(*R*)-1-(2-chloro-4-(((2-hydroxyethyl)amino)methyl)phenyl)ethoxy	21	7.6778
20	imidazo[1,2-*a*]pyridin-3-yl	(*R*)-1-(2-chloro-4-(((3-hydroxypropyl)amino)methyl)phenyl)ethoxy	19	7.7212
21	imidazo[1,2-*a*]pyridin-3-yl	(*R*)-1-(2-chloro-4-(((1-hydroxy-2-methylpropan-2-yl)amino)methyl)phenyl)ethoxy	23	7.6383
22	imidazo[1,2-*a*]pyridin-3-yl	(*R*)-1-(2-chloro-4-((4-fluoropiperidin-1-yl)methyl)phenyl)ethoxy	13	7.8861
23	imidazo[1,2-*a*]pyridin-3-yl	(*R*)-1-(2-chloro-4-((4-methylpiperazin-1-yl)methyl)phenyl)ethoxy	17	7.7696
24	imidazo[1,2-*a*]pyridin-3-yl	(*R*)-(1-(2-chloro-4-((4-hydroxypiperidin-1-yl)methyl)phenyl)ethoxy)	27	7.5686
25	imidazo[1,2-*a*]pyridin-3-yl	(*R*)-1-(2-chloro-4-((3-hydroxypyrrolidin-1-yl)methyl)phenyl)ethoxy	20	7.6990
26	imidazo[1,2-*a*]pyridin-3-yl	(*R*)- 1-(2-chloro-4-((3-oxopiperazin-1-yl)methyl)phenyl)ethoxy	12	7.9208
27	imidazo[1,2-*a*]pyridin-3-yl	(*R*)-1-(2-chloro-4-((1,1-dioxidothiomorpholino)methyl)phenyl)ethoxy	16	7.7959

28	imidazo[1,2-*a*]pyridin-3-yl	(*R*)-1-(4-((tert-butylamino)methyl)-2-fluorophenyl)ethoxy)	46	7.3372
29	imidazo[1,2-*a*]pyridin-3-yl	(*R*)-1-(4-((tert-butylamino)methyl)-2-methylphenyl)ethoxy	150	6.8239
30	imidazo[1,2-*a*]pyridin-3-yl	(*R*)-1-(4-((tert-butylamino)methyl)-2-cyclopropylphenyl)ethoxy	210	6.6778
31	imidazo[1,2-*a*]pyridin-3-yl	(*R*)-1-(4-((tert-butylamino)methyl)-2-(difluoromethyl)phenyl)ethoxy	20	7.6990
32	imidazo[1,2-*a*]pyridin-3-yl	(*R*)-1-(4-((tert-butylamino)methyl)-2-((difluoromethoxy)methyl)phenyl)ethoxy	9.8	8.0088

Table S2. The structures of 8-amino-4, 5-dihydro-1*H*-pyrazolo[4,3-*h*]quinazoline-3-carbaldehyde derivatives and their activities in Plk1

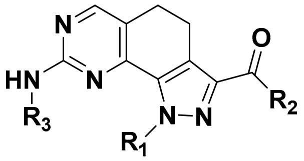

No.	Substiteuents			Activity (nM)	
	R^1	R^2	R^3	IC_{50}	pIC_{50}
33	Me	H	phenyl	68	7.1675
34	H	H	phenyl	248	6.6055
35	cyclohexyl	H	phenyl	143	6.8447
36	*i*Pro	H	phenyl	430	6.3665
37	Me	OH	Phenyl	110	6.9586
38	Me	NHMe	Phenyl	4215	5.3752
39	Me	NH_2	2-trifluoromethylphenyl	432	6.3645
40	Me	NH_2	3-trifluoromethylphenyl	51	7.2924
41	Me	NH_2	4-trifluoromethylphenyl	872	6.0695
42	Me	NH_2	2-acetylphenyl	346	6.4609
43	Me	NH_2	3-acetylphenyl	100	7.0000
44	Me	NH_2	4-acetylphenyl	197	6.7055
45	Me	NH_2	2-methyloxyphenyl	42	7.3768
46	Me	NH_2	3-methyloxyphenyl	135	6.8697
47	Me	NH_2	4-methyloxyphenyl	256	6.5918
48	Me	NH_2	2-methylthiophenyl	97	6.3116
49	Me	NH_2	2-(methylamino)phenyl	110	6.9586
50	Me	NH_2	2-fluorophenyl	125	6.9031
51	Me	NH_2	2-isopropylphenyl	365	6.4377
52	Me	NH_2	2-(methylcarboxy)phenyl	1117	5.9519
53	Me	NH_2	2-carbamoylphenyl	2076	5.6828
54	Me	NH_2	2-sulfamoylphenyl	3733	5.4279
55	Me	NH_2	[1,1'-biphenyl]-2-yl	1565	5.8055
56	Me	NH_2	2-phenoxyphenyl	278	6.5560
57	Me	NH_2	2-benzylphenyl	943	6.0255
58	Me	NH2	2-(phenylamino)phenyl	949	6.0227
59	Me	NH_2	2-benzoylphenyl	1969	5.7058
60	Me	NH_2	2-(phenylthio)phenyl	2033	5.6919
61	Me	NH_2	2-aminophenyl	150	6.8239
62	Me	NH_2	2-acetamidophenyl	2523	5.5981
63	Me	NH_2	2-acetyl-3-(4-methylpiperazin-1-yl)phenyl	2051	5.6880
64	Me	NH_2	2-acetyl-4-(4-methylpiperazin-1-yl)phenyl	464	6.3335
65	Me	NH_2	2-acetyl-5-(4-methylpiperazin-1-yl)phenyl	109	6.9626
66	Me	NH_2	2-methoxy-4-(4-methylpiperazin-1-yl)phenyl	40	7.3979

S2. Syntheses of 4-bromomethyl-3-chlorobenzyloxy (*t*-butyl)dimethylsilane

Scheme S1. Synthesis of 4-bromomethyl-3-chlorobenzyloxy (*t*-butyl)dimethylsilane.

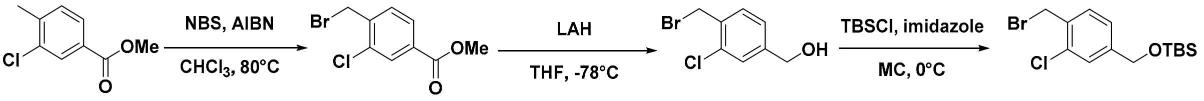

s1 s2 s3 s4 (C)

4-(Bromomethyl)-3-chlorobenzoate (s2)

Methyl 4-(bromomethyl)-3-chlorobenzoate (s1, 0.542 mmol) was dissolved in 2.71 ml of $CHCl_3$, AIBN (0.0542 mmol) and NBS (0.813 mmol) were sequentially added, followed by stirring at 80 °C for 20 hours. The reaction mixture was cooled to room temperature and concentrated *in vacuo*, followed by column chromatography and purification under EA : Hex (1:100) conditions to obtain methyl 4-(bromomethyl)-3-chlorobenzoate (s2; 70%). ^{1}H NMR (400 MHz, DMSO) δ 7.97 (d, *J* = 1.7 Hz, 1H), 7.90 (d, *J* = 1.7 Hz, 1H), 7.78 (d, *J* = 8.0 Hz, 1H), 4.79 (s, 2H), 3.87 (s, 3H).

(4-(Bromomethyl)-3-chlorophenyl)methanol (s3)

Compound s2 (0.372 mmol) was dissolved in 3.72 ml of THF, and Lithium aluminum hydride (0.223 mmol) was dropwise at -78 °C, followed by stirring for 1 hour. After completion of the reaction, work up was performed with ethyl acetate and 1N HCl solution. The organic layer was dried with anhydrous sodium sulfate (Na_2SO_4), and the solvent was evaporated, followed by column chromatography and purification under EA:Hex (1:5) conditions to obtain compound s3 (31%) ; ^{1}H NMR (400 MHz, DMSO) δ 7.56 (d, *J* = 7.8 Hz, 1H), 7.42 (s, 1H), 7.29 – 7.25 (m, 1H), 5.35 (t, *J* = 5.8 Hz, 1H), 4.73 (s, 2H), 4.50 (d, *J* = 5.8 Hz, 2H).

((4-(bromomethyl)-3-chlorobenzyl)oxy)(tert-butyl)dimethylsilane (s4)

Compound s3 (0.317 mmol) was dissolved in 1.59 ml of MC, TBSCl (0.476 mmol) and imidazole (0.634 mmol) were added, followed by stirring for 1 hour. After completion of the reaction, work up was performed with MC and H2O. The organic layer was dried with anhydrous sodium sulfate (Na_2SO4) and the solvent was evaporated to give compound s4 (99%).; ^{1}H NMR (400 MHz, DMSO) δ 7.58 (dd, *J* = 7.9, 2.1 Hz, 1H), 7.40 (d, *J* = 6.7 Hz, 1H), 7.29 (d, *J* = 7.0 Hz, 1H), 4.81 (s, 2H), 4.72 (s, 2H), 0.90 (s, 9H), 0.08 (s, 6H).

S3. Dose-response curve for IC_{50} evaluation of compound 15 (Reaction Biology Corp. *Kinase Hot Spot^{SM}* service)

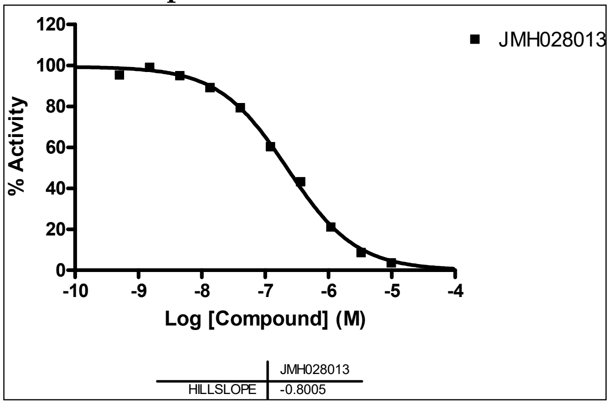

S4. Representative ^{1}H NMR spectrum

13a

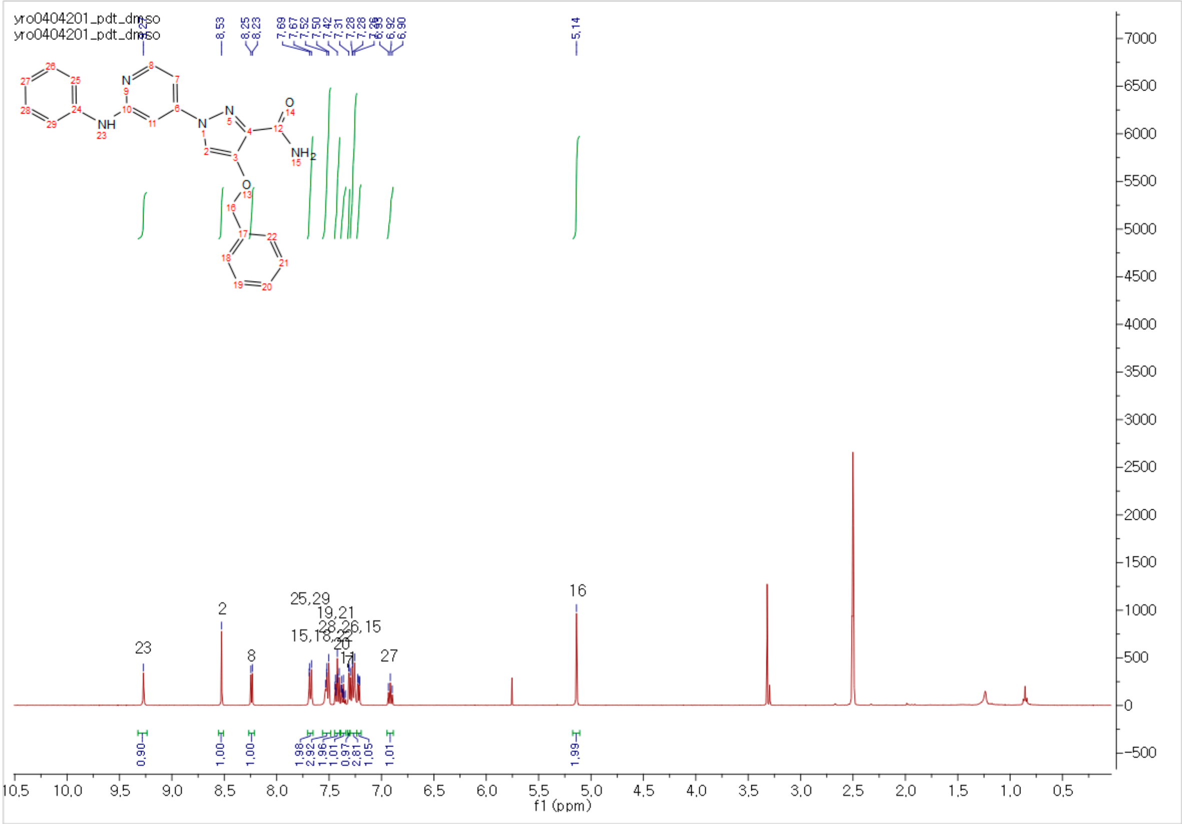

14a

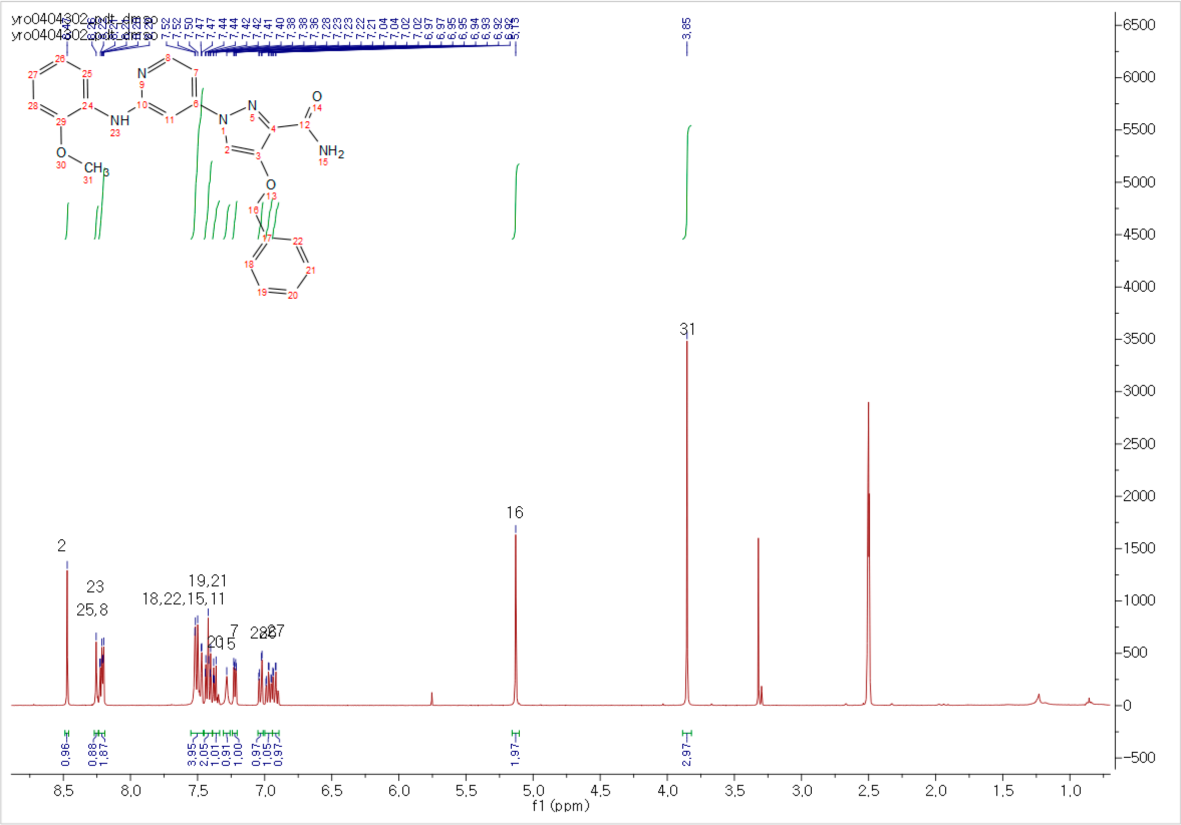

15

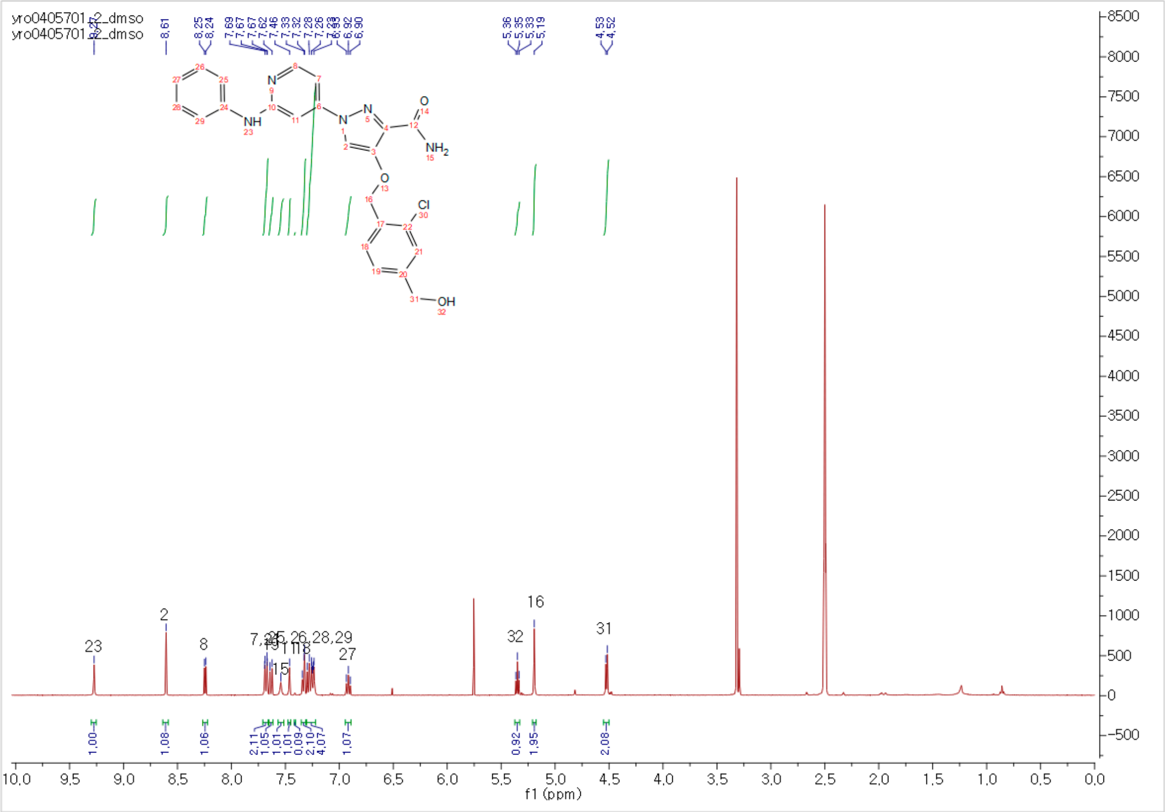

16

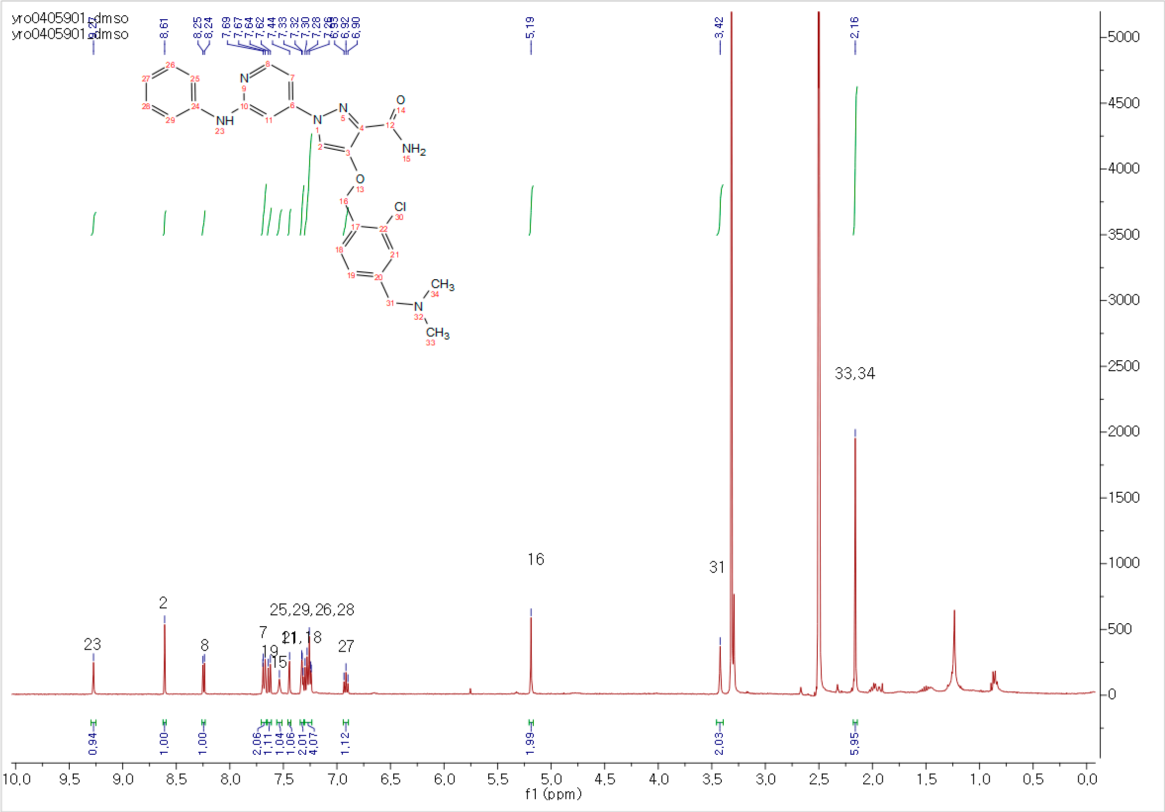

17

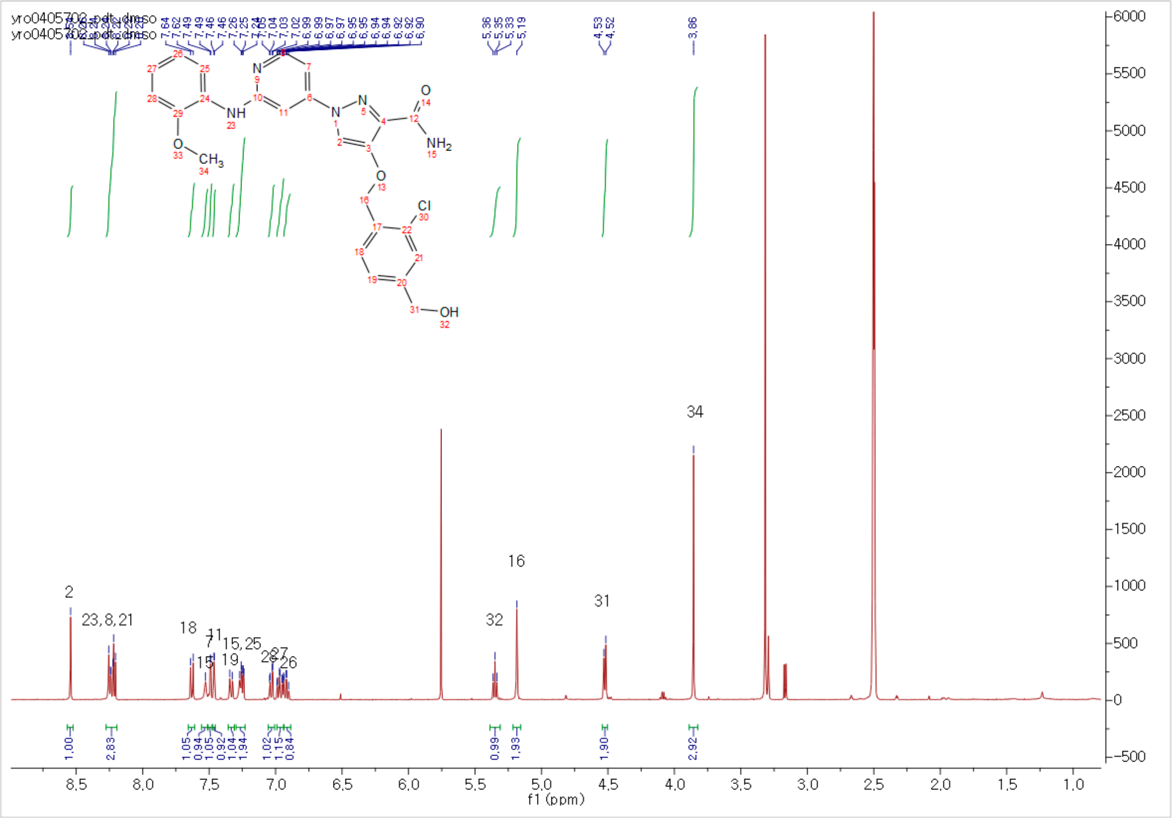

18

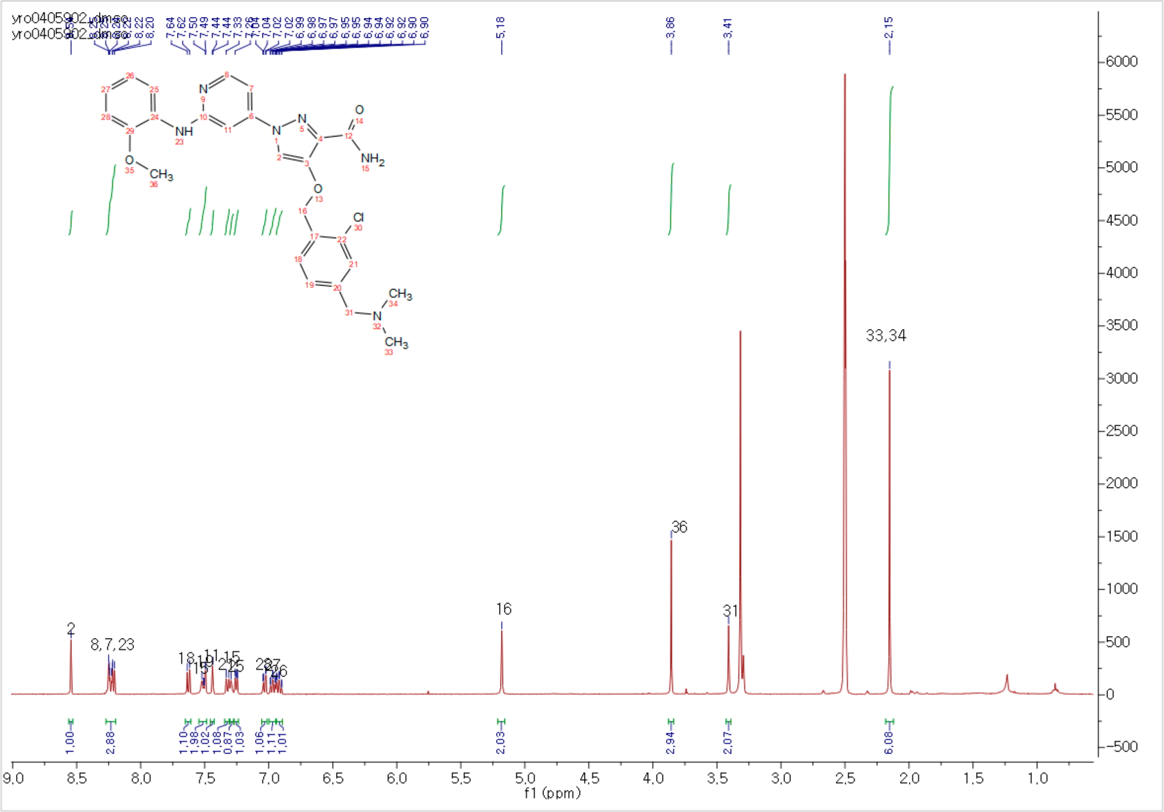

S5. Representative 13C NMR spectrum

8a

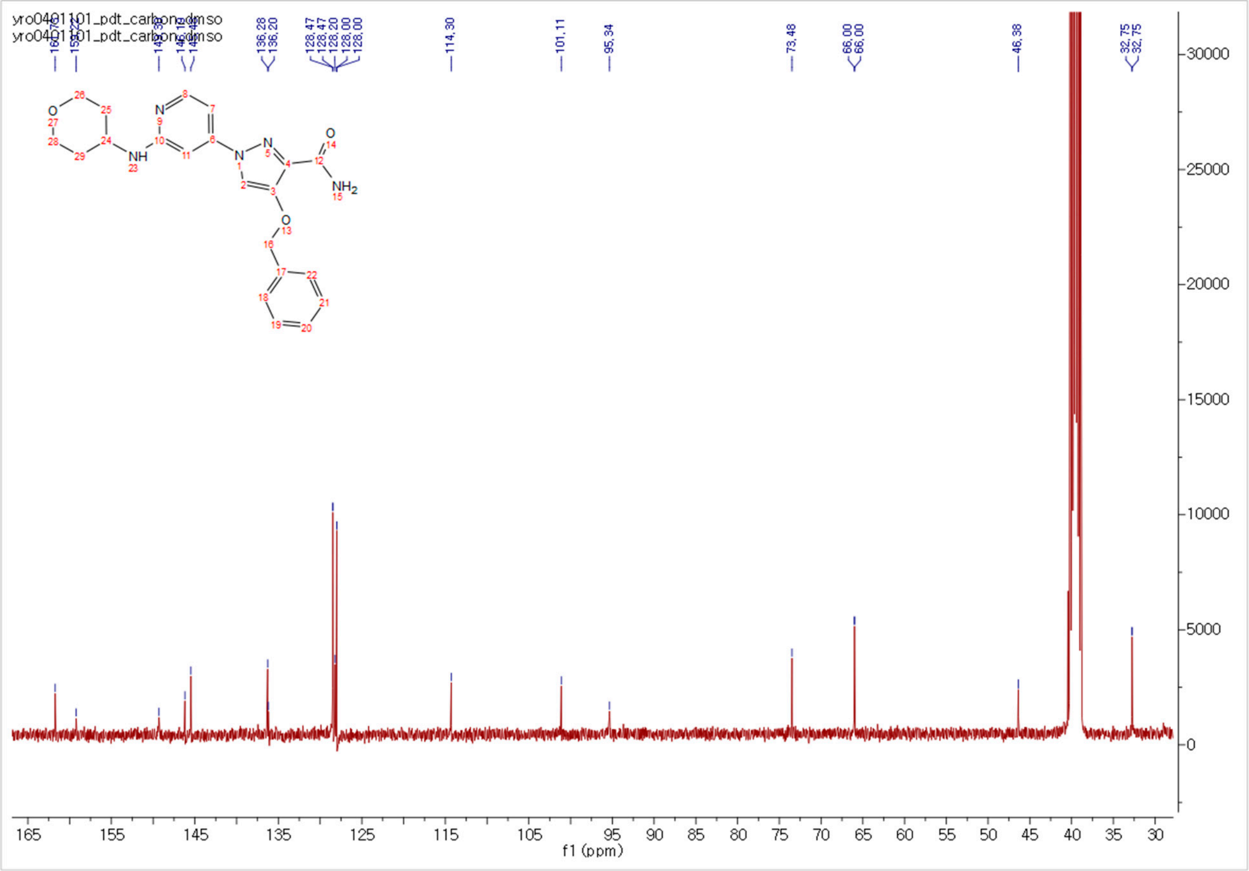

13a

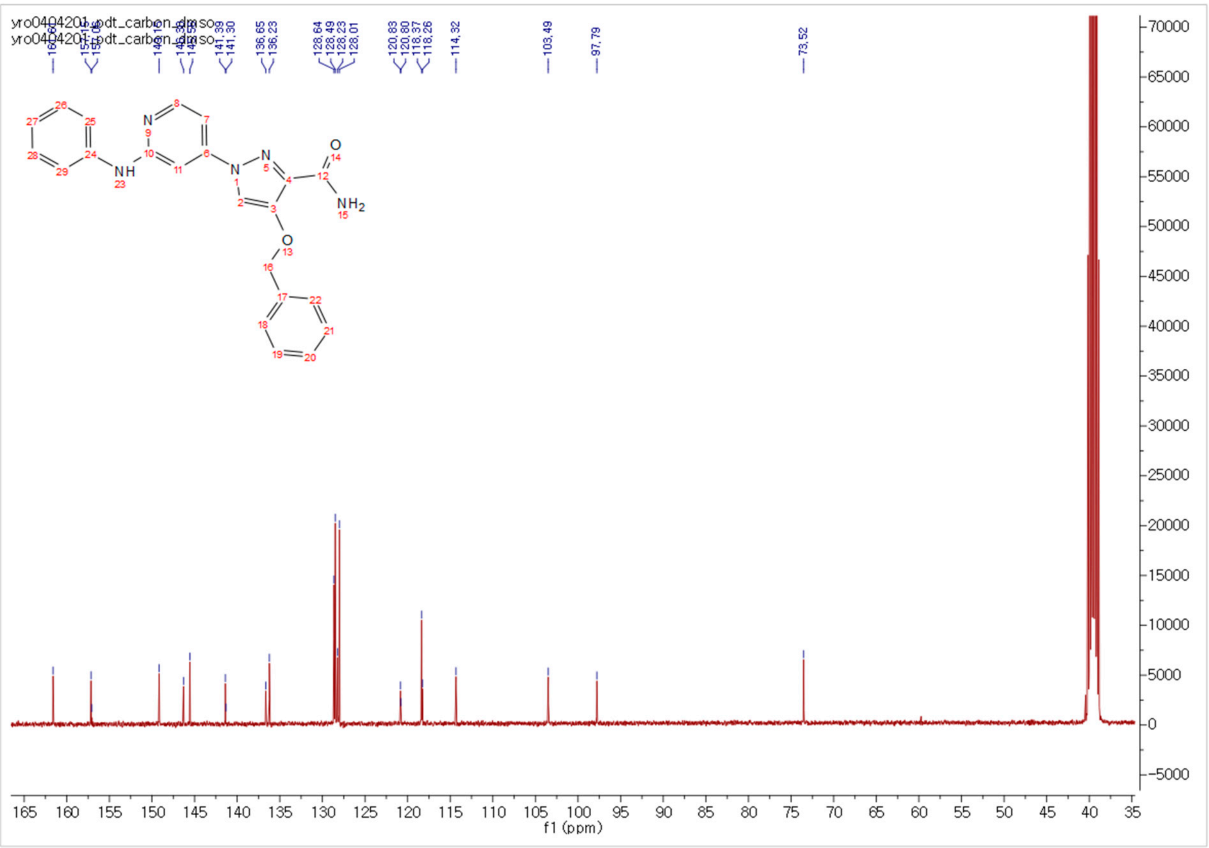

14a

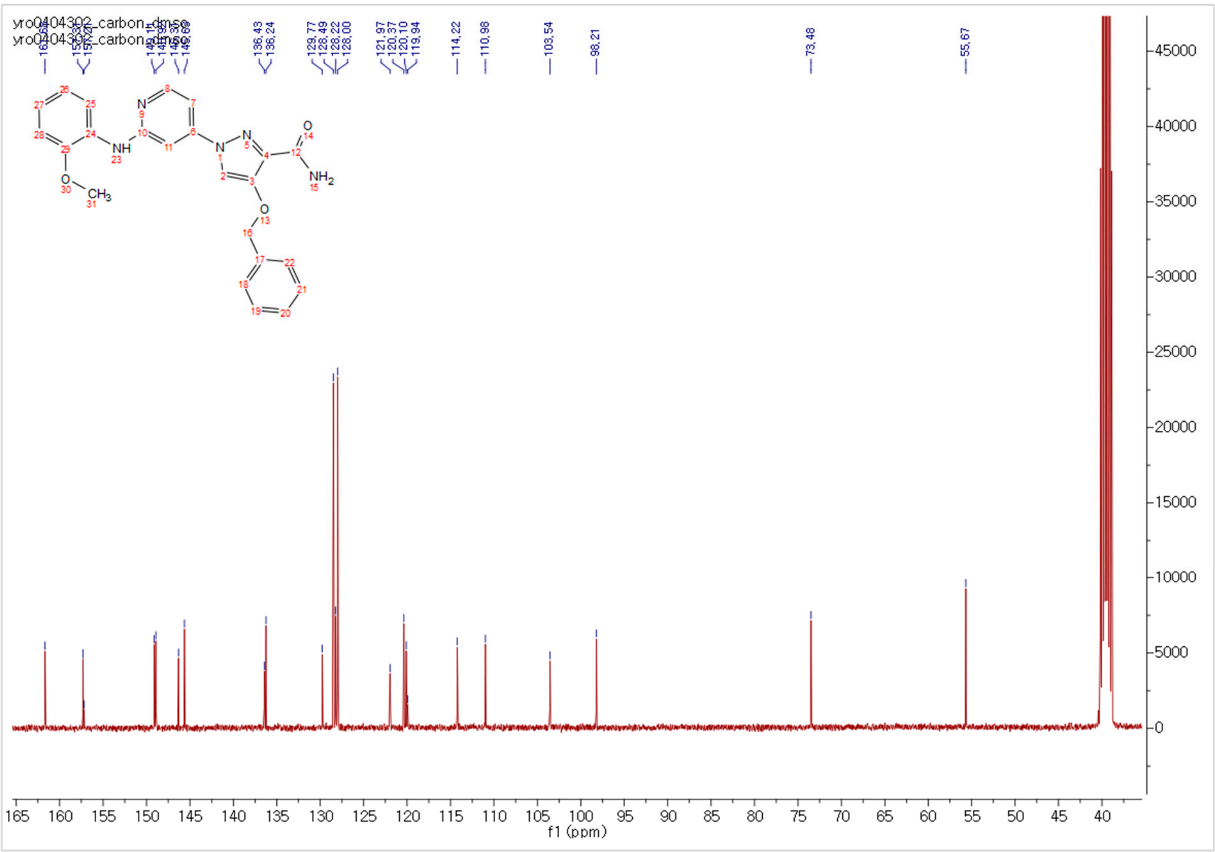

15

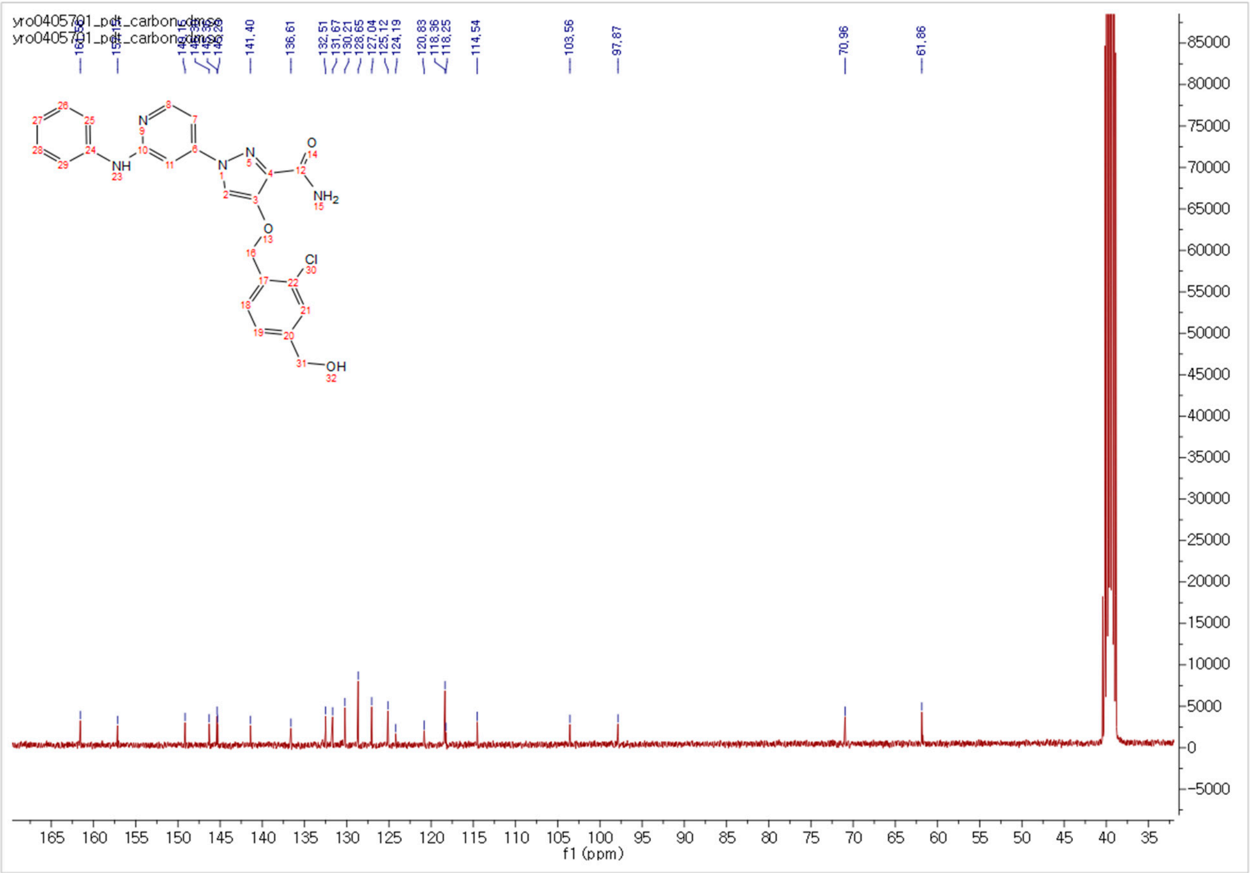

Supplement: Supplementary file 1 [file ijms-22-03865-s001.pdf]
